# A review of the internationalization of state-owned firms and sovereign wealth funds: Governments’ nonbusiness objectives and discreet power

**DOI:** 10.1057/s41267-022-00522-w

**Published:** 2022-05-11

**Authors:** Alvaro Cuervo-Cazurra, Anna Grosman, William L. Megginson

**Affiliations:** 1 D’Amore-McKim School of Business, Northeastern University, 360 Huntington Avenue, 313 Hayden Hall, Boston, MA 02115-5000 USA; 2 Loughborough University London, 3 Lesney Avenue, Here East, Queen Elizabeth Olympic Park, London, E20 3BS UK; 3 Michael F. Price College of Business, The University of Oklahoma, 307 W. Brooks, Suite 205B, Norman, OK 73019 USA; 4 University of International Business and Economics, Beijing, China

**Keywords:** state-owned firms, state-owned multinationals, sovereign wealth funds, government ownership, firm’s objectives, internationalization, state capitalism, international finance, international business, power, corporate governance, political connections, political bias

## Abstract

We review and bridge the literature on the internationalization of state-owned firms and sovereign wealth funds to provide a novel understanding of how government ownership affects foreign investments in three ways. First, we explain how state-owned firms and funds behave differently from private ones because they need to balance governments’ nonbusiness objectives and firms’ business goals. This results in competing predictions on whether government ownership helps or hinders internationalization due to particular nonbusiness objectives. Second, building on the review, we provide suggestions on how to extend research topics and theories of the firm by incorporating these nonbusiness objectives in the internationalization decisions in four areas: home government’s endowments, characteristics, and attitudes; host-country expansion’s support, influence, and impact; home- and host-country relationship conflicts, mediation, and disguising; and management’s orientation, opacity, and arbitrage. Third, we capture how governments may use state-owned multinationals and sovereign wealth funds to nudge host-country governments by introducing the concept of discreet power and the use of four strategies (recognition, values, development, and supremacy) to achieve it. This helps to outline the beginning of a unified approach to how governments use their foreign investments to achieve nonbusiness goals.

**Supplementary Information:**

The online version contains supplementary material available at 10.1057/s41267-022-00522-w.

## Résumé

Nous passons en revue et rapprochons la littérature de l'internationalisation des entreprises publiques et celle des fonds souverains afin d’apporter une nouvelle compréhension de la façon dont la propriété gouvernementale influence les investissements étrangers. Cette contribution se concrétise de trois manières. Premièrement, nous expliquons comment les entreprises et les fonds publics se comportent différemment des entreprises privées, parce qu’ils doivent trouver un équilibre entre les objectifs non commerciaux des gouvernements et les objectifs commerciaux des entreprises. Ce qui génère les prédictions opposées sur le rôle que joue la propriété gouvernementale: est-ce qu’elle aide ou entrave l'internationalisation en raison d’objectifs non commerciaux particuliers? Deuxièmement, nous appuyant sur la revue des littératures, nous élaborons les suggestions sur la manière d'étendre, via l’incorporation des objectifs non commerciaux aux décisions d'internationalisation, les thèmes de recherche et les théories de l'entreprise dans quatre domaines: les dotations, les caractéristiques et les attitudes du gouvernement du pays d'origine; le soutien, l'influence et l'impact de l'expansion du pays d’accueil; les conflits, la médiation et le déguisement dans les relations entre pays d'origine et pays d'accueil; et l'orientation, l'opacité et l'arbitrage de la direction. Troisièmement, nous essayons de saisir la manière dont les gouvernements peuvent utiliser les multinationales publiques et les fonds souverains pour influencer les gouvernements des pays d’accueil, et ce en introduisant le concept de pouvoir discret et l'utilisation de quatre stratégies (reconnaissance, valeurs, développement et suprématie) pour y parvenir. Cela permet d’esquisser le début d'une approche unifiée de la manière dont les gouvernements utilisent leurs investissements étrangers pour atteindre des objectifs non commerciaux.

## Resumen

Revisamos y unimos la literatura sobre la internacionalización de las empresas de propiedad estatal y los fondos soberanos para proporcionar una comprensión novedosa de cómo la propiedad gubernamental afecta las inversiones extranjeras de tres maneras. Primero, explicamos cómo las empresas y los fondos estatales se comportan de manera diferente a los privados porque necesitan equilibrar los objetivos no comerciales de los gobiernos y los objetivos comerciales de las empresas. Esto da lugar a predicciones contradictorias sobre si la propiedad gubernamental ayuda o dificulta la internacionalización debido a objetivos particulares no comerciales. En segundo lugar, sobre la base de la revisión, proporcionamos sugerencias sobre cómo ampliar los temas de investigación y las teorías de la empresa mediante la incorporación de estos objetivos no comerciales en las decisiones de internacionalización en cuatro áreas: recursos, características y actitudes del gobierno local; el apoyo, la influencia y el impacto de la expansión del país anfitrión; conflictos, mediación y encubrimiento de las relaciones entre el país de origen y el país anfitrión; y la orientación, opacidad y arbitraje de la gestión. En tercer lugar, capturamos cómo los gobiernos pueden usar multinacionales estatales y fondos soberanos para empujar a los gobiernos de los países anfitriones mediante la introducción del concepto de poder discreto y el uso de cuatro estrategias (reconocimiento, valores, desarrollo y supremacía) para lograrlo. Esto ayuda a delinear el comienzo de un enfoque unificado sobre cómo los gobiernos utilizan sus inversiones extranjeras para lograr objetivos no comerciales.

## Resumo

Revisamos e ligamos a literatura sobre a internacionalização de empresas estatais e fundos soberanos para fornecer uma nova compreensão de como a propriedade do governo afeta investimentos estrangeiros de três formas. Em primeiro lugar, explicamos como empresas e fundos estatais se comportam de maneira diferente de entidades privadas porque precisam equilibrar objetivos não comerciais de governos e metas de negócios das empresas. Isso resulta em previsões antagônicas sobre se a propriedade do governo ajuda ou atrapalha a internacionalização devido a certos objetivos não comerciais. Em segundo lugar, com base na revisão, fornecemos sugestões sobre como estender tópicos de pesquisa e teorias da firma incorporando esses objetivos não comerciais nas decisões de internacionalização em quatro áreas: dotações, características e atitudes do governo local; apoio, influência e impacto da expansão do país anfitrião; conflitos, mediação e disfarce nas relações entre país de origem e anfitrião; e orientação, opacidade e arbitragem da administração. Em terceiro lugar, capturamos como governos podem usar multinacionais e fundos soberanos estatais para estimular governos de países anfitriões, introduzindo o conceito de poder discreto e o uso de quatro estratégias (reconhecimento, valores, desenvolvimento e supremacia) para alcançá-lo. Isso ajuda a delinear o início de uma abordagem unificada de como governos usam seus investimentos estrangeiros para atingir objetivos não comerciais.

## Abstract (zh)

我们对有关国有企业和主权财富基金国际化的文献进行了综述和链接, 以提供对政府所有权如何以三种方式影响外国投资的新理解. 首先, 我们解释国有企业和基金的行为与私营企业有何不同, 因为它们需要平衡政府的非商业目标和企业的商业目标. 这导致了关于政府所有权由于特定的非商业目标的原因是否有助于或阻碍国际化的相互竞争的预测. 其次, 在文献综述的基础上, 我们为如何通过将这些非商业目标纳入四个领域的国际化决策来扩展研究主题和公司理论提供了建议: 母国政府的禀赋, 特点和态度; 东道国扩张的支持、影响和影响力; 母国-东道国关系的冲突, 调解和掩饰; 以及管理层导向, 不透明度和套利. 第三, 我们确定政府是如何利用国有跨国公司和主权财富基金来推动东道国政府的, 这是通过引入谨慎权力的概念和使用四种策略 (认可, 价值, 发展和优越) 来实现的. 这有助于概述政府如何使用其外国投资实现非商业目标的统一方法的开始.

## Introduction

There has been a resurgence of state capitalism in international business, through which governments are increasingly becoming foreign investors. The 1980s and 1990s were a period of retrenchment for state capitalism as communist countries transitioned towards capitalism, and advanced and developing economies underwent deregulation and privatization (Megginson & Netter, 2001). However, despite these processes, governments continue to be significant investors. On the one hand, state-owned firms continue to exist and have grown, even if governments are no longer the sole owners. Thus, among the 100 largest publicly traded firms, 25 are state-owned, accounting for USD4.3 trillion in total revenues (Fortune, 2020). On the other hand, governments have increasingly created and funded sovereign wealth funds, which have undertaken a global investment spree. The top ten sovereign wealth funds have USD6.7 trillion in assets under management (Global SWF, 2020).

However, although governments own both state-owned multinationals and sovereign wealth funds and can coordinate their behavior, as illustrated in the opening quote, research has studied these firms separately, creating a theoretical gap. Studies on state-owned multinationals focus on their foreign direct investments, usually appear in international business journals, and tend to study country and entry mode selection (Cuervo-Cazurra & Li, 2021; Cuervo-Cazurra, Inkpen, Musacchio, & Ramaswamy, 2014), while analyses of sovereign wealth funds focus on their foreign portfolio investments, are commonly published in finance and economics journals, and tend to study profitability (Bortolotti, Fotak, & Megginson, 2015; Dewenter, Han, & Malatesta, 2010). Recent research has initiated a cross-fertilization, for example, analyzing the implication of state ownership on stock returns in multinationals (Karolyi & Liao, 2017) or country and entry mode selection of sovereign wealth funds (Bertoni & Lugo, 2013; Knill, Lee, & Mauck, 2012). Despite this, most studies treat them as independent of one another, limiting the theorization on the role of governments as foreign investors.

Hence, in this article, we bridge these two streams of research, which have operated in parallel, to provide a novel understanding of how governments act as foreign investors in three ways. First, we explain how state-owned firms and funds differ from private ones in their internationalization because they need to balance governments’ nonbusiness objectives and firms’ business goals. This results in competing arguments, with some discussing restrictions on foreign expansion, while others highlight support for foreign investments by influencing host-country governments. Second, building on the review, we provide suggestions on how to extend research topics and theories of the firm by incorporating these nonbusiness objectives in the internationalization decision in four areas: home government’s endowments, characteristics, and attitudes; host-country expansion’s support, influence, and impact; home- and host-country relationship conflicts, mediation, and disguising; and management’s orientation, opacity, and arbitrage. Third, to capture how governments may use state-owned multinationals and sovereign wealth funds to nudge host-country governments, despite their differences in the level of control over investee firms, we introduce the concept of *discreet power* as the beginning of a unified approach to how governments use their foreign investments to achieve nonbusiness goals.

These ideas contribute to two lines of research: state ownership and internationalization. On the one hand, foreign investments by state-owned multinationals and sovereign wealth funds question the traditional theoretical justifications for firms’ state ownership as solving market imperfections and enabling development (Lawson, 1994). Under this logic, governments should not invest abroad because addressing those imperfections is the task of host-country governments. Thus, the international expansion of state-owned firms and funds highlights the need to consider governments’ nonbusiness objectives as complements to the traditional market imperfection explanations. On the other hand, governments’ foreign investments question the focus on profitability as the ultimate driver of internationalization traditionally assumed (Buckley & Casson, 1985), as state-owned entities also pursue nonbusiness objectives internationally (Cuervo-Cazurra et al., 2014). This requires a rethinking of the theoretical predictions on the selection and management of foreign investments, as governments may use state-owned multinationals and sovereign wealth funds as tools for achieving nonbusiness objectives abroad by exercising discreet power. This new concept of discrete power additionally contributes to the literature by providing a bridge between the soft and hard power concepts from political economy (Nye, 2004).

## Governments as Investors

### A Brief History of Governments as Investors

State ownership and control of the economy have oscillated between reforms and reversals (Cuervo-Cazurra, Gaur, & Singh, 2019). During much of the twentieth century, governments were active investors. The Great Depression and the Second World War led most governments in advanced countries to become active investors to facilitate reconstruction. In communist countries, state ownership expanded, driven by an ideology of state control of the means of production. Developing countries increased government ownership of industries considered necessary to facilitate industrialization. Governments created sovereign wealth funds to invest excess funds from natural resources (Aguilera, Capapé, & Santiso, 2016), while some state-owned firms became multinationals (Vernon, 1979).

The economic crises of the 1970s questioned the effectiveness of government ownership and state control of the economy, resulting in large privatizations in the 1980s and 1990s. Even sectors considered to be the exclusive realm of the state, such as utilities or defense, underwent privatization. Studies explained the privatization objectives, decision, timing, and process (Megginson, Nash, Netter, & Poulsen, 2004), as well as the performance of the newly privatized firms (Boubakri & Cosset, 1998). However, many of these were partial privatizations, with governments retaining stakes in firms considered strategic.

The 2000s led to another rethinking of state ownership. Governments in advanced markets responded to the Great Recession of 2007–2009 by supporting, and in some cases nationalizing, firms considered essential. Meanwhile, the success of the Chinese government in managing the recession and the foreign expansion of its state-owned firms showed that state capitalism was again a viable development model. Thus, the literature evolved from assuming that state ownership was harmful to considering its positive effects (Grosman, Okhmatovskiy, & Wright, 2016; Mazzucato, 2011; Wright, Wood, Musacchio, Okhmatovskiy, Grosman, & Doh, 2021). Studies on state-owned multinationals analyzed recent phenomena, such as state ownership diversity (Cuervo-Cazurra et al., 2014; Inoue, Lazzarini, & Musacchio, 2013).

### Governments as Investors: Direct and Indirect Ownership

These processes generated a wide diversity of types of government ownership of firms, which Table 1 summarizes. We study the internationalization of two types of state-owned entities: state-owned firms and sovereign wealth funds. State-owned firms are enterprises over which the government has significant ownership and control; even minority stakes allow the government to influence decisions (Chen, Musacchio, & Li, 2019; Inoue et al., 2013). They are created by the state or emerge from the nationalization of private firms, and they are sometimes grouped into state holding companies, such as SASAC in China and Temasek in Singapore. Sovereign wealth funds are government investment vehicles commonly funded by foreign exchange assets or natural resource wealth and managed separately from official reserves (Kimmitt, 2008). They tend to invest in assets to obtain commercial returns (Balding, 2011). Their small investment stakes do not typically provide control over the invested firms, but they can nevertheless exercise influence as active investors. We do not study public pension funds, whose main source of funds is pension contributions and whose objectives are social, even if they invest abroad and in some cases in private equity funds (Kimmitt, 2008; Preqin, 2017).

**Table 1:** Types of state-owned businesses

	Direct ownership				Indirect ownership		
	State entity/ agency	State (fully) owned firm	State majority-owned firm	State minority-owned firm	Sovereign wealth fund	Public pension fund	State bank loaned firm
Legally separate firm	No	Yes	Yes	Yes	Yes	Yes	Yes
Budget	No separate budget	Separate budget	Separate budget	Separate budget	Separate budget	Separate budget	Separate budget
State ownership	Direct ownership	Direct ownership	Direct ownership	Direct ownership	Indirect via ownership by sovereign wealth fund	Indirect via ownership by state-owned pension fund	Indirect via convertible loan by state-owned bank
Level of state ownership	Full ownership	Full ownership	Majority ownership	Minority ownership and/or golden share in a private company	Minority investment in private firm by sovereign wealth fund	Minority investment in private firm by state pension fund	Minority investment in private firm via convertible loan by state-owned bank
Types of managers	Civil servants	Civil servants/ professional managers	Civil servants/ professional managers	Professional managers	Professional managers	Professional managers	Professional managers
Level of government influencing firm	Central/ federal	Central/ federal; province/ state; municipal/ city	Central/ federal; province/ state; municipal/ city	Central/ federal; province/ state; municipal/ city	Central/ federal	Central/ federal; province/ state; municipal/ city	Central/ federal; province/ state; municipal/ city
Type of investment abroad	–	Foreign direct investment	Foreign direct investment	Foreign direct investment	Foreign portfolio investment; foreign private equity investment	Foreign portfolio investment; foreign private equity investment	Foreign portfolio investment
Drivers of foreign investment (traditional)	–	Markets Inputs/factors of production Strategic assets	Markets Inputs/ factors of production Strategic assets	Markets Inputs/ factors of production Strategic assets	Financial returns Diversification Stabilization	Financial returns Diversification	Financial returns Diversification
Drivers of foreign investment (nontraditional)	–	Diplomacy Influence National development	Diplomacy Influence National development Independence	Diplomacy Influence National development	Diplomacy Influence	Diplomacy Influence	Diplomacy Influence

*Source*: Adapted and extended from Cuervo-Cazurra et al. (2014)

Governments at multiple levels can own both types of state entities. Among state-owned firms, most companies owned by lower-tier governments are not international, but some are, like the automobile manufacturer Volkswagen, which is part-owned by the German state of Lower Saxony. Among sovereign wealth funds, the conventional view is that they are owned by the central government (hence the term sovereign), but lower-level governments also own them, like the Alaska Permanent Fund Corporation.

### Differences in Objectives of State and Private Investors: Business and Nonbusiness Goals

There are significant differences in goals between state and private investors. At the core of the theory of the firm lies its objective function, which is maximizing profits or market returns (Friedman, 1988) and protecting shareholder rights (Shleifer & Vishny, 1997). These goals also apply to state-owned entities. However, most state entities are charged with achieving additional nonbusiness goals that conflict with profit maximization. For state-owned firms, nonbusiness goals include politically motivated investments in innovation, employment, or social stability (Lazzarini, Mesquita, Monteiro, & Musacchio, 2020; Sun, Deng, & Wright, 2020). For sovereign wealth funds, nonbusiness goals include diversification of domestic markets, stabilization, sustainable investments for social goals, and protection from corrupt elites (Bernstein, Lerner, & Schoar, 2013; Knill et al., 2012).

In Table 2, we clarify these differences in goals between private and state-owned investors. Our view is that, rather than having a dichotomy of objectives, with private investors interested in business objectives and state-owned ones in nonbusiness objectives, they differ in the relative importance of the goals. Governments are not passive owners uninterested in performance, but rather active shareholders seeking multiple objectives. For example, sovereign wealth funds target strategic industries (Nowacki & Monk, 2017) and are used as part of governments’ external investments (Truman, 2007).

**Table 2:** Objective functions of private and state-owned internationalized businesses

	Shareholder objectives			Stakeholders’ objectives				Government objectives	
	Profit Maximization	Optimization via shareholder activism	Corporate social responsibility	Sustainable/ethical portfolio investment	Employment generation in the home country	Industrial policy and innovation in the home country	Protect inter-generational wealth in the home country	Diversification and stabilization of the home country	Diplomatic influence
Private multinational	✓✓✓	✗	✓	✗	✗	✗	✗	✗	✗
Private fund with foreign investments	✓✓✓	✓✓	✗	✓	✗	✗	✗	✗	✗
State-owned multinational	✓	✗	✓	✗	✓✓	✓✓	✗	✗	✓✓
Sovereign wealth fund with foreign investments	✓	✓	✗	✓✓	✓	✓	✓✓	✓✓	✓✓

### Differences in Corporate Governance of State and Private Investors

State-owned entities follow hybrid governance. The governance practices of state-owned entities are usually considered deviations from the accepted standards for the governance of private firms (OECD, 2015a), and the recommendation is to make the governance of state-owned entities resemble as much as possible the governance of private firms (Shleifer, 1998). However, state-owned entities are hybrid organizations in their corporate governance, using elements of private corporate governance to enhance monitoring with elements of public administrative governance to enhance accountability. They use governance tools differently or employ additional instruments to develop a suitable corporate governance structure (Okhmatovskiy, Grosman, & Sun, 2021). This hybrid governance is reflected in governance guidelines, board structures, and nontraditional governance mechanisms adopted by state-owned entities.

#### Guidelines

Guidelines for state-owned firms and sovereign wealth funds reflect the particularities of government ownership. These differ in objectives from the codes of good governance for private firms, which are usually designed to address the separation of ownership and control (Aguilera & Cuervo-Cazurra, 2004). State-owned firms can follow the Organization for Economic Co-operation and Development (OECD) guidelines (OECD, 2015b). If they are publicly traded, they must adhere to listing regulations, such as disclosure of ownership and executive compensation, board independence, and required board structures (OECD, 2013). Sovereign wealth funds can follow corporate governance practices, commonly known as the Santiago Principles (IWGSWF, 2008). These are voluntary standards for investment practices, corporate governance, transparency, and accountability.

#### Boards

The board of directors is a universal governance mechanism for advising and monitoring managers (John & Senbet, 1998), but its composition and functions in state-owned entities differ from those in private firms. In state-owned entities, board nominations are usually the government’s responsibility (OECD, 2013), resulting in appointments without transparent and competitive selection procedures. Boards are commonly composed of civil servants, tasked with pursuing the public interest, and are increasingly also composed of independent directors. However, although the latter have incentives to generate financial results (Grosman, Aguilera, & Wright, 2019), they still have indirect affiliations to politicians pursuing noneconomic objectives (Menozzi, Gutiérrez Urtiaga, & Vannoni, 2012). These boards undergo frequent replacements, mirroring election cycles rather than performance (Kuzman, Talavera, & Bellos, 2018).

Boards of state-owned firms perform their functions differently to reflect the requirements of state ownership. They monitor whether managerial actions are aligned both with the business goals and the public mission of state-owned firms (Okhmatovskiy et al., 2021). Their resource provision function differs because government support isolates them from financial pressure. Finally, their strategic function is fulfilled directly by the state (OECD, 2018), which is sometimes suboptimal because it is biased by political goals (Lazzarini & Musacchio, 2015).

#### Nontraditional governance mechanisms

State-owned entities sometimes employ governance mechanisms rarely used in private firms that enable the government to impose its preferences without securing the agreement of other shareholders. Okhmatovskiy et al. (2021) highlight four mechanisms. First, governments use performance contracts to establish expectations regarding outcomes that state-owned firms commit to deliver (OECD, 2015a). These contracts provide boards with greater autonomy in decision-making without sacrificing the goals of the government as a shareholder. Second, through loan agreements from state-owned banks, governments can pursue a politically driven industrial policy agenda. Third, state-owned firms are under informal influence from government officials in matters ranging from issuing permits to choosing suppliers for state-funded projects or providing support for internationalization. Finally, political elites influence managers of state entities through administrative mechanisms. For example, in China, the influence of the Communist Party permeates economic and social activities, leading Chinese state-owned entities to be called “hybrid business–political actors” (Lin & Milhaupt, 2013).

### Governments as Foreign Investors

We study how these differences in objectives and governance of state and private investors affect internationalization, helping advance the theorization of international business and state ownership through cross-fertilization. By bringing insights from one topic to the other, we outline the beginning of a unified theory of how governments use their foreign investments to achieve nonbusiness goals. This complements previous studies that separately analyzed sovereign wealth funds (Aguilera et al., 2016; Megginson & Fotak, 2015; Megginson & Gao, 2020) and state-owned multinationals (Cuervo-Cazurra et al., 2014; Rygh, 2019).

## Internationalization of State-Owned Multinationals and Sovereign Wealth Funds

The systematic review and analysis of the content of past studies on the internationalization of state-owned firms and sovereign wealth funds reveals new insights into the role of governments’ nonbusiness objectives. Online Appendix A provides the research design and lists the articles reviewed.

### Evolution of the Literature and Phenomenon

#### The internationalization of state-owned firms

The literature on state-owned multinationals evolves through three phases. It starts in the 1970s and 1980s with a few theoretical and interview-based studies on the growing expansion of state-owned multinationals, usually from advanced economies (Aharoni, 1982; Mazzolini, 1979). However, the extensive privatization processes of the 1990s redirect interest toward the privatization of state-owned multinationals (Dewenter & Malatesta, 2001; Djankov & Murrell, 2002). In the 2000s, there is a rediscovery of the topic, and, by the late 2010s, there is a significant expansion in the number and variety of studies. There is also a change from studying only state-owned multinationals to comparing them to privately-owned multinationals, facilitating the identification of the uniqueness in their behavior.

This evolution of the literature reflects the transformation of state-owned firms. Table 3 illustrates the evolution of state ownership in selected countries from 1970 to 2017. The general trend is a sharp decline in state ownership between 1970 and 1995 and an increase in the 2010s. The most notable drop is among transition economies, as expected from their move from communism to capitalism, but there is also a significant drop elsewhere. Table 4 presents the top 25 publicly traded state-owned firms. The list is dominated by Chinese firms, but there are state-owned firms from other countries, including advanced ones.

**Table 3:** State ownership around the world

	1970	1975	1980	1985	1990	1995	2000	2005	2010	2015	2019
*Advanced economies*											
Australia	2.48	2.48	2.48	2.14	1.52	0.81	0.81	0.81	0.81	2.53	2.51
Belgium	3.28	3.28	2.65	2.65	1.83	1.83	1.83	1.83	1.83	1.56	1.99
Canada	2.44	2.44	2.44	2.44	2.44	2.44	2.44	2.44	2.44	1.46	2.79
Denmark	3.22	3.22	3.22	3.22	3.22	3.22	3.22	3.22	3.18	3.04	2.53
France	4.30	4.30	4.30	5.99	1.92	2.56	2.56	2.56	2.56	2.56	2.84
Germany	1.99	1.99	1.99	1.99	1.98	0.72	0.00	0.00	0.89	0.00	1.39
Italy	4.62	4.62	4.62	4.22	3.93	0.36	0.36	0.36	0.36	0.48	2.20
Japan	1.84	1.84	1.84	1.84	0.81	0.81	0.81	0.81	1.21	0.61	0.63
Netherlands	2.31	2.31	2.31	2.11	2.11	2.38	2.38	2.38	2.44	3.35	2.24
Norway	3.32	3.32	3.32	3.32	3.32	3.32	3.51	3.51	3.51	3.22	2.92
Singapore	6.16	6.16	6.16	6.16	6.16	6.16	6.16	6.16	6.16	6.16	5.63
South Korea	3.46	3.46	3.12	3.14	2.34	2.07	3.21	3.21	3.21	2.59	2.78
Spain	4.29	4.29	2.58	2.12	2.12	2.12	1.58	1.22	1.26	1.84	2.22
Sweden	3.01	3.01	3.01	3.01	3.01	1.68	1.68	1.68	1.68	1.80	2.23
Switzerland	2.20	2.20	2.02	2.02	1.80	1.80	0.84	0.00	0.00	1.02	0.96
Taiwan	4.07	4.07	4.07	4.07	3.54	2.97	2.97	2.97	1.92	1.92	2.51
United Kingdom	5.09	5.09	2.47	1.73	1.73	1.29	1.29	1.29	1.28	1.75	1.71
United States	2.06	2.06	1.24	1.24	1.24	1.24	1.24	0.00	0.00	0.00	0.69
*Transition economies*											
Albania	10.00	10.00	10.00	10.00	10.00	3.46	1.85	2.00	1.78	2.17	1.47
China	10.00	10.00	7.95	7.01	7.01	6.09	6.29	6.29	6.46	6.46	6.03
Hungary	10.00	9.89	9.89	4.48	1.44	1.24	1.24	1.24	1.88	4.99	4.66
Kazakhstan	10.00	10.00	10.00	10.00	5.47	3.96	3.51	4.55	4.53	5.28	4.69
Poland	5.74	5.74	5.74	5.74	3.61	3.05	2.30	2.30	2.30	2.91	3.11
Romania	10.00	10.00	10.00	10.00	6.42	5.23	3.67	3.28	2.75	3.22	2.70
Russia	10.00	10.00	10.00	10.00	10.00	4.66	5.41	5.63	5.57	6.37	5.42
Serbia	8.24	8.24	8.24	7.61	6.50	4.71	3.47	2.66	3.33	3.06	2.90
Ukraine	10.00	10.00	10.00	10.00	10.00	5.99	4.20	3.18	2.27	1.80	3.38
Vietnam	10.00	10.00	10.00	10.00	6.47	5.34	5.34	5.57	5.57	4.05	5.26
*Developing economies*											
Argentina	4.57	4.57	4.41	4.53	2.98	1.31	1.61	2.26	2.95	3.01	2.76
Bangladesh	10.00	5.14	4.80	4.80	4.46	4.46	4.46	4.46	4.46	3.81	3.52
Brazil	4.96	4.96	4.87	4.87	4.19	2.94	2.94	2.94	2.94	2.94	1.82
Chile	2.16	2.17	2.17	2.17	2.17	1.55	1.55	1.55	1.55	1.02	1.60
Colombia	3.27	3.27	3.27	3.27	3.27	1.82	1.82	1.82	1.82	2.86	2.20
Egypt	4.92	4.98	4.93	4.93	4.47	3.86	3.86	5.65	5.65	8.67	6.91
India	4.80	4.46	4.46	4.46	4.46	3.27	3.27	3.27	3.27	3.27	1.89
Indonesia	6.09	6.09	6.09	6.09	6.09	6.09	3.58	3.58	3.66	2.89	4.14
Iran	6.67	7.55	7.55	7.83	6.97	6.97	6.97	7.07	7.07	6.86	5.86
Malaysia	3.99	4.77	4.77	5.49	5.49	5.49	5.49	5.49	5.49	6.04	5.30
Mexico	6.22	6.22	6.22	4.04	3.51	3.03	3.03	3.29	3.07	1.56	2.73
Nigeria	6.81	6.81	6.83	6.83	5.31	5.31	4.43	4.43	4.98	3.84	3.92
Pakistan	6.43	8.39	5.45	5.45	4.93	3.49	3.49	3.49	4.03	3.90	4.33
Philippines	4.26	5.58	5.58	5.58	2.87	1.79	1.62	1.62	2.61	0.98	2.51
Qatar	7.77	7.74	7.27	7.27	7.31	7.31	6.16	6.16	6.16	6.80	7.17
Saudi Arabia	7.30	7.30	7.63	7.63	7.63	7.63	8.07	8.07	8.05	8.36	8.33
South Africa	2.82	2.82	2.82	2.82	2.41	2.41	1.77	1.77	2.55	1.84	1.87
Thailand	3.96	3.96	3.96	3.96	2.73	2.46	2.44	3.11	3.11	3.60	3.67
Turkey	5.49	5.49	4.50	3.43	3.08	3.08	3.08	2.79	2.36	3.46	3.50
UAE	10.00	8.57	8.55	8.55	8.55	8.54	8.56	5.49	5.49	5.55	5.55
Venezuela	2.58	5.11	5.11	5.11	4.71	4.14	5.22	6.49	5.90	6.82	7.96

All data from the Fraser Institute, Economic Freedom of the World: 2021 Annual Report, available at fraserinstitute.org, accessed November 3, 2021. We have selected representative countries from each category of development where there is variation in state ownership levels over time. The state ownership data are based on the index of state ownership constructed by the Fraser Institute, which is equal to (*V*_*I*_ − *V*_min_)/(*V*_max_ − *V*_min_) multiplied by 10; where *V*_*I*_ is the country’s state ownership score, while the *V*_max_ and *V*_min_ are set at 2.5 standard deviations above and below the average, respectively. We transformed the index so that 0 represents less state ownership and 10 represents more ownership (by subtracting 10 and multiplying the result by − 1)

**Table 4:** Top 25 state-owned firms by revenue

Rank	Global rank	Name	Country	Revenues, USD B	Profit, USD B	Assets, USD B	Employees, K
1	2	Sinopec Group	China	407.0	6.8	317.5	582.6
2	3	State Grid	China	383.9	8.0	596.6	907.7
3	4	China National Petroleum	China	379.1	4.4	608.1	1,344.4
4	6	Saudi Aramco	Saudi Arabia	329.8	88.2	398.3	79.0
5	7	Volkswagen	Germany	282.8	15.5	547.8	671.2
6	18	China State Construction Engineering	China	205.8	3.3	294.1	335.0
7	21	Ping An Insurance	China	184.3	21.6	1180.5	372.2
8	24	Industrial & Commercial Bank of China	China	177.1	45.2	4322.5	445.1
9	30	China Construction Bank	China	158.9	38.6	3651.6	370.2
10	35	Agricultural Bank of China	China	147.3	30.7	3571.5	467.6
11	43	Bank of China	China	135.1	27.1	3268.8	309.4
12	45	China Life Insurance	China	131.2	4.7	648.4	180.4
13	50	China Railway Engineering Group	China	123.3	1.5	153.0	302.4
14	52	SAIC Motor	China	122.1	3.7	121.9	151.8
15	53	Fannie Mae	USA	120.3	14.2	3503.3	7.5
16	54	China Railway Construction	China	120.3	1.4	155.6	364.9
17	55	Gazprom	Russia	118.0	18.6	352.4	473.8
18	60	Japan Post Holdings	Japan	109.9	4.4	2647.3	245.5
19	62	Nippon Telegraph and Telephone	Japan	109.4	7.9	213.0	319.0
20	64	China National Offshore Oil	China	108.7	7.0	184.9	92.1
21	65	China Mobile Communications	China	108.5	12.1	266.2	457.6
22	76	Rosneft Oil	Russia	96.3	10.9	208.5	335.0
23	78	China Communications Construction	China	95.1	1.3	232.1	197.3
24	79	China Resources	China	94.8	3.6	232.3	396.5
25	86	Deutsche Telekom	Germany	90.1	4.3	191.6	210.5

*Source*: Data from Fortune (https://fortune.com/global500/2020/) accessed on September 13, 2020. The list includes only publicly traded firms

*B* billion, *K* thousands

#### The internationalization of sovereign wealth funds

Research on the internationalization of sovereign wealth funds is limited and recent, with studies on this topic only appearing in the 2010s. One reason is that, although some sovereign wealth funds were founded in the middle of the twentieth century, most were created only after 2000 (Aguilera et al., 2016). They have expanded abroad in search of diversification, especially those from small economies unable to absorb surpluses. The investments have moved from passive to active ones as governments intervene in global financial markets to seek political, social, and financial objectives (Monk, 2011; Wood & Wright, 2015).

Although sovereign wealth funds were created more recently than state-owned firms, they have grown much faster. Table 5 provides statistics on sovereign wealth funds and public pension funds by country. China leads the ranking in terms of total assets under management, followed by Norway and Abu Dhabi. Their growth is driven by the accumulation of wealth from natural resources, especially oil, and foreign exchange reserves by central banks, especially after the 1998 East Asian financial crisis. Table 6 lists the 25 largest sovereign wealth funds, ranked by assets under management. The ranking is led by Norway’s Government Pension Fund Global, China Investment Corporation, and the Abu Dhabi Investment Authority. The listing shows great variety in the country of origin, creation date, and size.

**Table 5:** Sovereign wealth funds around the world

Country	Number of sovereign wealth funds	Sovereign wealth funds’ assets (USD bn)
*Advanced economies*		
Australia	5	275
Canada	2	14
Denmark	0	4
Finland	1	8
France	1	34
Japan	1	0
Netherlands	0	0
Norway	2	1,187
Singapore	2	821
South Korea	1	157
Sweden	0	0
UK	0	0
USA	22	230
*Transition economies*		
Bulgaria	0	0
China	8	2,269
Kazakhstan	4	145
Poland	0	0
Russia	2	169
Turkmenistan	1	1
Uzbekistan	1	15
*Developing economies*		
Argentina	0	0
Bahrain	2	19
Brazil	0	0
Chile	2	21
Colombia	2	19
India	1	2
Indonesia	0	0
Jordan	0	0
Kuwait	3	574
Malaysia	2	37
Mexico	1	7
Peru	1	5
Philippines	0	0
Oman	3	48
Qatar	1	345
South Africa	1	2
Saudi Arabia	2	819
Thailand	0	0
Turkey	1	34
UAE - Abu Dhabi	3	1,005
UAE - Dubai	3	354

*Source* Data from Global SWFs (www.globalswf.com) accessed on 17 July 2020

**Table 6:** Top sovereign wealth funds by assets under management

Rank	Name	Country	Founding	Assets under management, USD B
1	Norway Government Pension Fund Global	Norway	1990	1,108.7
2	China Investment Corporation	China	2007	940.6
3	Abu Dhabi Investment Authority	Abu Dhabi, UAE	1976	579.6
4	Kuwait Investment Authority	Kuwait	1953	533.7
5	Hong Kong Monetary Authority Investment Portfolio	Hong Kong	1935	528.1
6	GIC Private Limited	Singapore	1981	453.2
7	Temasek Holdings	Singapore	1974	417.4
8	Public Investment Fund	Saudi Arabia	1971	390.0
9	National Council for Social Security Fund	China	2000	325.0
10	Investment Corporation of Dubai	Dubai, UAE	2006	305.2
11	Qatar Investment Authority	Qatar	2005	295.2
12	Mubadala Investment Company	Abu Dhabi, UAE	2002	232.2
13	Brunei Investment Agency	Turkey	2016	222.3
14	National Welfare Fund	Russia	2008	165.4
15	Korea Investment Corporation	Korea	2005	157.3
16	Future Fund	Australia	2006	110.6
17	National Development Fund of Iran	Iran	2011	91.0
18	Alberta Investment Management Corporation	Canada	2008	86.3
19	Alaska Permanent Fund Corporation	USA	1976	67.3
20	Samruk-Kazyna	Kazakhstan	2008	63.1
21	Kazakhstan National Fund	Kazakhstan	2000	61.1
22	Brunei Investment Agency	Brunei	1983	60.0
23	Libyan Investment Authority	Libya	2006	60.0
24	University of Texas Investment Management Company	USA	1996	48.4
25	Texas Permanent School Fund	USA	1854	46.5

*Source*: Data from SWFI (https://www.swfinstitute.org/fund-rankings/sovereign-wealth-fund) accessed on September 13, 2020

Sovereign wealth funds are increasingly large investors in private equity, even if historically they held portfolios primarily composed of public equities and debt. Sixty-one percent of sovereign wealth funds directly hold private equity in their portfolios (Preqin, 2017). This is driven by the search for higher returns and a growing sophistication of funds (Nowacki & Monk, 2017). There are significant cross-country variations, however. Middle Eastern and Asian sovereign wealth funds account for the largest share of funds investing in private equity, while none of the Latin American sovereign wealth funds invest in private equity (Preqin, 2017). Investing in private equity also helps sovereign wealth funds achieve the mission of developing their local economies. Some sovereign wealth funds were specifically created to coinvest in private equity to help domestic firms abroad or to attract long-term foreign capital into their home economies (e.g., Bahrain’s Mumtalakat Holding). These two activities increasingly come together in bilateral partnerships in which investments support targeted industries’ development (Nowacki & Monk, 2017).

#### Building bridges

As shown above, there is accelerating growth in the size and internationalization of both state-owned multinationals and sovereign wealth funds. Our analysis suggests that national governments tend to specialize in the vehicles they use to internationalize investments. Small and wealthy emerging countries and advanced economies tend to invest in the rich world primarily through sovereign wealth funds. In contrast, large emerging economies seem to channel their international investments through state-owned firms to other emerging countries or occasionally advanced economies. China seems to be an exception, using both state-owned firms and sovereign wealth funds to invest everywhere.

### Theoretical Foundations

#### The internationalization of state-owned firms

Most studies build on a variety of theories to explain the internationalization of state-owned firms. Among those studies that use a single theoretical approach, there appears to be a theoretical dichotomy in predictions, with some theories highlighting the advantage and others the disadvantage of stateness (Cuervo-Cazurra & Li, 2021; Musacchio, Monteiro, & Lazzarini, 2019). On the one hand, studies using agency theory point out the additional costs that state-owned firms incur as a result of their multilevel agency problems, which hampers internationalization. Similarly, studies building on neo-institutional theory highlight how state-owned firms suffer from lower legitimacy in host countries that limits entry. On the other hand, studies that draw on the resource-based view argue that state-owned firms can access state resources and thus internationalize more widely. There remains a need to increase the impact and sharpness of these arguments by clarifying the predictions of the theories on internationalization. More depth can be added by identifying the conditions, like the level of state ownership (Kalasin, Cuervo-Cazurra, & Ramamurti, 2019), under which the arguments of one theory compensate for the predictions of another.

#### The internationalization of sovereign wealth funds

Most studies on sovereign wealth funds focus on their rise and the influences on their investment strategies and returns, rather than on developing theory. Explanations are mainly based on principles of optimal portfolio allocation, attempting to identify whether sovereign wealth funds show biases in their investment decisions because they are state owned. One set of studies investigates how governments use foreign investments by sovereign wealth funds to promote national development (Haberly, 2014; Kamiński, 2017; Sun, Li, Wang, & Clark, 2014). Another set with links to political economy focuses on the location choice of sovereign wealth funds’ foreign investments, and how political relationships with host countries influence the location and amounts invested (Johan, Knill, & Mauck, 2013; Knill et al., 2012; Makhoul, Musacchio, & Lazzarini, 2020). The few studies in management use a mix of theoretical foundations, like transaction costs, neo-institutional, or signaling theory (Aguilera et al., 2016; Vasudeva, Nachum, & Say, 2018).

#### Building bridges

The two research streams show a diversity of theoretical bases. In contrast to analyses of private investors, studies on governments as foreign investors tend to build links, sometimes implicitly, to political economy theory to explain the role that the government plays in decisions. They can extend theories by challenging some of the assumptions on which they have been built, namely private investors searching for returns, by incorporating nonbusiness objectives in the theorization.

### Empirical Bases

#### The internationalization of state-owned firms

While the early literature analyzed European state-owned multinationals, recent studies usually focus on Chinese firms, with Brazilian firms coming in a distant second. One reason might be that the size and growth of these two economies helped their state-owned firms reach the scale and funds needed for internationalization. Moreover, in both countries, governments implemented an active policy of supporting outward foreign direct investment in an apparent desire to have national champions that dominate strategic industries (Lazzarini, Musacchio, Bandeira-de-Mello, & Marcon, 2015; Luo, Xue, & Han, 2010). These support policies are unusual elsewhere, likely biasing our understanding of the role of governments in the internationalization of state-owned firms. Despite their importance, we know little about leading state-owned multinationals from advanced economies or resource-rich countries. Early studies analyzed samples containing only state-owned multinationals (Mazzolini, 1980), but recent ones usually compare them to private firms (Li, Xia, & Lin, 2017). Few analyses include state-owned firms from multiple countries, limiting our understanding of home-country drivers of internationalization, such as the type of capitalism (Mariotti & Marzano, 2019).

#### The internationalization of sovereign wealth funds

Research on sovereign wealth funds tends to be dichotomous, analyzing either many sovereign wealth funds from multiple countries or only one. The former studies rely on publicly traded firms in which sovereign wealth funds have invested, and collect data on all available sovereign wealth funds regardless of country of origin. Some match investments by sovereign wealth funds and pension funds to understand the role of the government in investment decisions (Boubakri, Cosset, & Grira, 2016). This may bias our view of their investment strategies, as we know little about investments in private equity and illiquid assets like real estate. The latter studies focus on investments by one fund, usually Norway’s Government Pension Fund Global (Vasudeva, 2013; Vasudeva et al., 2018) or Singapore’s Temasek (Gnabo, Kerkour, Lecourt, & Raymond, 2017; Phelps, 2007), because these are transparent.

#### Building bridges

The research databases used appear to bias the knowledge that we are gaining on governments as foreign investors. Most studies rely on datasets of publicly traded firms, which are only partially state owned. Future research can add novelty by using datasets of wholly state-owned firms and investments that are not publicly traded, and by extending the countries researched beyond Brazil and China in studies of state-owned multinationals and Norway and Singapore in analyses of sovereign wealth funds.

### Patterns of Internationalization

#### The internationalization of state-owned firms

The literature on the internationalization of state-owned firms has studied a wide variety of dimensions of internationalization and, with some exceptions (Aharoni, 1986), seems to agree that the government plays a significant role in the foreign expansion of state-owned firms. However, the specifics of such influence are under debate.

We separate the studies into categories based on the type of relationships on which they focus: internationalization motive, internationalization level, and the selection of country of destination and entry mode. The main lessons are that state-owned multinationals (1) have politically influenced nonbusiness motives; (2) select more challenging host countries and use more acquisitions, even if the market reactions are negative; and (3) show conflicting behavior on the level of internationalization, with some studies proposing a higher level and others a lower one (Cuervo-Cazurra & Li, 2021).

First, among studies on *internationalization motives*, some research argues that state-owned multinationals behave differently, although they do not compare them to private firms. These articles indicate that state-owned firms are driven to internationalize by political motives, such as the desire for governmental access to strategic assets and natural resources (Buckley, Cross, Tan, Xin, & Voss, 2008). Other studies indicate that managers of state-owned firms drive internationalization in search of independence from government control (Choudhury & Khanna, 2014). Other studies that compare state-owned and private firms find that the government influences both state and private firms, and that they pursue similar internationalization objectives (Ren, Manning, & Vavilov, 2019). This conflict in arguments may result from the Chinese government’s internationalization mandate and support to all firms (Luo et al., 2010). For example, to facilitate cross-border investments by both state-owned and private firms, the Chinese government is establishing foreign trade agreements with host countries (Buckley, Clegg, Voss, Cross, Liu, & Zheng, 2018).

Second, state ownership seems to have a competing influence on the *level of internationalization*. Some studies argue that the agency problems of state-owned firms lead to less internationalization (Li, Xia, Shapiro, & Lin, 2018; Mazzolini, 1979), while others propose that the provision of preferential resources to state-owned firms supports their internationalization (Luo et al., 2010). The literature that compares state-owned and private firms shows conflicting patterns of findings. Some find that state-owned firms have more foreign investments thanks to their government support (Benito, Rygh, & Lunnan, 2016; Ramasamy, Yeung, & Laforet, 2012), while others show that they have fewer investments due to home-country overdependence (Deng, Yan, & van Essen, 2018; Huang, Xie, Li, & Reddy, 2017), and yet others indicate that they are similar to private firms (Hu & Cui, 2014), especially state-owned firms with strong home-country governance (Estrin, Meyer, Nielsen, & Nielsen, 2016).

A third group of articles investigates the *selection of the country of investment and entry mode*, acknowledging that governments’ nonbusiness objectives affect the decisions. Governments direct state-owned firms to countries with good diplomatic relationships and that are similar to the home country in the weakness of their institutions (Zhang & He, 2014), because they can influence the host country to support and protect their firms. Entry modes are influenced by state ownership, which helps to achieve more control over foreign operations (Dikova, Panibratov, & Veselova, 2019; Kalotay & Sulstarova, 2010). Among entry modes, most studies analyze acquisitions. In this case, state-owned firms execute more acquisitions than private firms and gain full ownership thanks to their home-government support (Li et al., 2017; Meyer, Ding, & Li, 2014). However, there are conflicting market reactions to acquisitions. Some propose adverse reactions to acquisitions because of the perceived higher inefficiency of state-owned firms (Chen & Young, 2010; Li et al., 2018), while others argue for positive reactions from the preferential government treatment (Du & Boateng, 2015). Some studies argue that state-owned firms prefer purchasing stand-alone assets instead of firms, and that their choices are similar to private firms when their home country has well-functioning institutions (Grøgaard, Rygh, & Benito, 2019).

Fewer studies have analyzed other dimensions of internationalization besides foreign direct investments, such as how state ownership affects offshore outsourcing, imports, or exports (Cuervo-Cazurra & Dau, 2009). One way to clarify the conflicting arguments on whether the government helps or hinders internationalization is to identify the conditions under which such relationships hold, such as the attitudes or types of governments, the level of development of the country, or the industry of operation.

#### The internationalization of sovereign wealth funds

The sparse literature on the internationalization of sovereign wealth funds can be grouped into two sets: studies on their *behavior*, including investment portfolio strategy and decision making, and analyses that *compare the investment performance of sovereign wealth funds to private funds*. The general conclusion from these studies is that (1) sovereign wealth funds invest abroad in search of diversification and behave differently from private funds, and (2) they invest in better firms and countries with stronger investor protection.

Sovereign wealth funds invest abroad to diversify their macroeconomic and political exposures by investing in global equities and debt, especially those of developed economy firms. This stream of research on portfolio selection unanimously finds that sovereign wealth funds behave differently, as they invest in firms in strategic industries (Boubakri et al., 2016; Haberly, 2011; Sun et al., 2014), in countries with strong legal institutions and higher economic growth (Debarsy, Gnabo, & Kerkour, 2017), and in large, profitable, international firms (Karolyi & Liao, 2017; Megginson & Fotak, 2015; Mietzner, Schiereck, & Schweizer, 2015). Sovereign wealth funds from OECD countries invest differently from those from non-OECD countries (Avendaño & Santiso, 2011), use different target selection criteria, depending on whether or not the target is from an OECD country, and tend to re-invest in the same country (Candelon, Sy, & Arezki, 2011).

Some analyses explore how differences between the home and the host country affect investments. They indicate that sovereign wealth funds are used by their governments as tools to promote national economic development by investing abroad in priority sectors (Haberly, 2011; Kamiński, 2017; Sun et al., 2014). Other studies find that investments through sovereign wealth funds’ portfolio companies get the right level of managerial attention if these investments represent an important portion of the overall portfolio (Makhoul et al., 2020).

Sovereign wealth funds also seek to influence host-country governments. This influence can be achieved by providing money to campaign finance firms, where allowed, especially in industries with more restrictions on foreign investments (Calluzzo, Dong, & Godsell, 2017), or by having representatives on the board in energy firms (Kamiński, 2017). Policy makers in host countries can also actively court sovereign wealth fund investments in local firms as long-term patient capital or to expand overseas market opportunities for these firms, and to politically reach out to sovereign wealth fund-owning governments to advance their interests (Haberly, 2011, 2014; Lavelle, 2017; Thatcher & Vlandas, 2016). Bortolotti et al. (2015) and Bahoo, Alon, and Paltrinieri (2020) summarize research on the target selection and decision-making processes of sovereign wealth funds.

Comparisons of sovereign wealth fund and private fund investment performance study their cross-border strategies to understand whether they behave differently in terms of choice (private or public equity investments), risk appetite, and returns. Particular attention is given to sovereign wealth funds’ transparency and their target firms’ performance (Bortolotti et al., 2015; Dewenter et al., 2010; Karolyi & Liao, 2017; Megginson, 2017).

It remains unclear why some sovereign wealth funds decide to remain opaque, even though they may face challenges and restrictions by doing so when investing overseas. Further, there is still a substantial gap in the literature on how macro-issues, such as the characteristics of the governments or economies, or micro-factors, such as corporate governance, affect the internationalization motives of sovereign wealth funds.

#### Building bridges

The arguments and findings of studies of governments as foreign investors confirm that they behave differently from private investors. State ownership appears to play a dual role in the internationalization of state-owned firms and sovereign wealth funds by constraining and supporting internationalization. This reflects the tension between the disadvantage and advantage of stateness (Cuervo-Cazurra & Li, 2021; Musacchio et al., 2019). On the one hand, state-owned investors seem to have a lower tendency to go abroad because of governments’ preference for domestic investments to gain political support, and because of the increased scrutiny and constraints that state-backed investments face in host countries. On the other hand, once they decide to invest abroad, they benefit from the government’s financial and diplomatic support, targeting countries and projects that are too risky for private investors.

## Research Agenda: The Way Forward

Despite the significant progress made in our understanding of the government as a foreign investor, many areas can benefit from more research. Building on this review, we suggest several research avenues that integrate our understanding of the internationalization of state-owned multinationals and sovereign wealth funds further. We organize them by topic and detail research questions in Table 7.

**Table 7:** Future research questions to answer in the study of state-owned multinationals and sovereign wealth funds

Topic	Suggested research questions
Home Government: endowments, characteristics, and attitudes	
Endowments	Which country characteristics lead to the creation and internationalization of a state-owned multinational/sovereign wealth fund?
	What country characteristics help a state-owned multinational/sovereign wealth fund work better in achieving business or nonbusiness objectives abroad?
	Which channels are the most effective for home-country governments in using state-owned multinationals/sovereign wealth funds to facilitate the development of the domestic economy through foreign expansion?
	How do international operating strategies of state-owned multinationals/sovereign wealth funds evolve over time/with the level of economic development/in response to economic development in home countries?
Characteristics	How do a political regime and its change in the government affect the international strategies of state-owned multinationals/sovereign wealth funds?
Attitudes	Why do some governments exert control on the foreign expansion of state-owned multinationals/sovereign wealth funds, while others give them autonomy so that they internationalize like private firms?
Host-country expansion: support, influence, and impact	
Support	What is the effect of government ownership and support on the riskiness of foreign investments by sovereign wealth funds and state-owned multinationals?
	What are the coordination mechanisms among foreign investments from the same industry by sovereign wealth funds and state-owned multinationals that reinforce state influence in that industry abroad?
	How do industry characteristics of host countries (technological orientation, regulation, or level of competition) affect the internationalization and performance of state-owned multinationals/sovereign wealth funds?
Influence	How do governments as foreign investors affect host-country governments?
	How do state-owned multinationals/sovereign wealth funds address market imperfections abroad?
	How do the political and commercial expertise of the top management team and board affect the internationalization and performance of state-owned multinationals/sovereign wealth funds in comparison to private funds/ multinationals?
	How does CEO succession affect the internationalization strategy of state-owned multinationals/sovereign wealth funds?
	How does the ideology of the state-owned multinationals/sovereign wealth funds affect the behavior of managers of foreign subsidiaries/ foreign-invested firms?
	How do the political connections and ideology of top management teams affect the foreign strategy and performance of state-owned multinationals/sovereign wealth funds?
	How do the strategies of state-owned multinationals/sovereign wealth funds change industry characteristics in host countries?
Impact	How can host governments manage the trade-offs between the benefits of investments by state-owned multinationals/sovereign wealth funds and the costs of interference by the home government of state-owned multinationals/sovereign wealth funds?
Home- and host-country relationships: conflicts, mediation, and disguising	
Conflicts	How can governments use evaluations and other informal political restrictions to limit hostile governments’ foreign investments?
	What are the institutional varieties in responses to foreign investments by state-owned multinationals and sovereign wealth funds?
Mediation	How can state-owned multinationals/sovereign wealth funds reduce the negative impact of conflict between home and host countries?
	How do changes in bilateral relations between home and host countries affect the strategies and performance of state-owned multinationals/sovereign wealth funds?
Disguising	How do host-country governments or citizens react to state-owned multinationals/sovereign wealth funds entry?
	How do state-owned multinationals/sovereign wealth funds respond to these reactions?
	How do the different strategies of discreet power of home governments affect host countries?
Management: orientation, opacity, and arbitrage	
Orientation	How does the internationalization of state-owned multinationals affect the degree of their efficiency, budget constraints, and decision-making independence?
	How does the time horizon of state-owned multinationals/ sovereign wealth funds affect the time horizon of strategic decision-making of managers in foreign subsidiaries/ foreign target firms?
Opacity	How do sovereign wealth fund corporate governance characteristics lead to better foreign investment performance relative to private funds?
	What are the mechanisms to mitigate foreign shareholder expropriation through sovereign wealth funds?
Arbitrage	How does the management coordination of foreign subsidiaries of state-owned multinationals/foreign targets of sovereign wealth funds differ from foreign subsidiaries of multinational companies or foreign-invested firms of private funds?
	How do the strategies of foreign subsidiaries of state-owned multinationals/foreign targets of sovereign wealth funds differ from foreign subsidiaries of multinational companies or foreign-invested firms of private funds?
	How do the characteristics of state-owned multinationals/sovereign wealth funds affect internationalization in comparison to private funds or private multinational companies?
	How do sovereign wealth funds decide on their foreign portfolio allocations among the different asset classes in comparison to private funds?

### Home Government: Endowments, Characteristics, and Attitudes

From the review, it is apparent that home-country characteristics drive the internationalization of both state-owned multinationals and sovereign wealth funds. Sovereign wealth funds have been created in countries with strong economic growth and an excess of foreign exchange reserves and wealth accumulated through natural resources. State-owned multinationals have also been formed in interventionist states with a traditionally active role of the government, supporting the internationalization of home state-owned firms. We outline below what appear to be promising ideas.

#### Endowments

One fundamental question is the identification of how country endowments induce the creation of state-owned multinationals and sovereign wealth funds. The level of home-country exposure to financialization (Wood & Wright, 2013) can affect the choice of organizational form of the investment vehicle (e.g., countries with developed financial markets are more likely to have sovereign wealth funds). National governments appear to specialize in the vehicles they use to internationalize investments, with small but affluent economies investing exclusively through sovereign wealth funds, while bigger, emerging countries channel most of their international investments through state-owned firms. This can help solve the question of why some governments set up sovereign wealth funds from natural resources windfalls, while others spend the windfalls subsidizing the welfare of the population. Attitudes towards savings and consumption may explain this. Home-country endowments can also affect the level of internationalization. For instance, state-owned firms in some emerging markets receive abundant state financing for internationalization.

#### Characteristics

The characteristics of the political system of the home country is another interesting topic. Future research can analyze how political regimes and election cycles affect the international strategies of state-owned multinationals and sovereign wealth funds. One way is to separate between authoritarian and democratic governments. In democratic systems, the rotation of parties in power with diverging attitudes towards state government control of the economy can lead to sharp changes in the internationalization strategy of state-owned multinationals and sovereign wealth funds. Non-democratic systems do not face such rotation, which may enable state-owned businesses to adopt longer-term strategies. It would also be interesting to analyze the buffers that state-owned entities can create to reduce disruption to their long-term strategies with changes in government. A new government may question the decisions taken by a previous one regarding foreign state-owned entities. Finally, there is room for analysis of the use of lobbyists and campaign donations by foreign state-owned multinationals and sovereign wealth funds to influence host governments and their internationalization.

#### Attitudes

Future research could benefit from a more structured analysis of the role of the home government’s attitudes towards the internationalization of state-owned multinationals and sovereign wealth funds. For instance, research could use classifications of countries by the level of intervention in the economy (Wright et al., 2021) and identify patterns of foreign investments. This will reduce the criticism of the existing literature, especially on state-owned multinationals, as being based on a small set of countries, which limits generalization. Going deeper into the attitudes of the government towards its role in the economy, and not just the quality of institutions or the type of political system, can yield valuable insights. For example, one important question is why some governments control the foreign expansion of state-owned firms and sovereign wealth funds (e.g., China), while others give them autonomy (e.g., Norway). Politicians’ view of their role in the economy and the level of trust or contract enforceability may drive these differences.

### Host Country Expansion: Support, Influence, and Impact

How private multinationals make their selection of host countries and decide on the entry modes they use in these countries is well known (Rugman, 2009). In state-owned multinationals and sovereign wealth funds, there are new issues to consider.

#### Support

One of the core insights on internationalization is how home-country governments play a dual role in internationalization: they constrain the foreign expansion of state-owned entities by directing them towards political objectives at home, but, once those firms go out abroad, they support their expansion with state resources. This support from the government enables them to undertake investments that are too risky for most private investors and mitigates some of the risks (Rose, 2009). Sovereign wealth funds can benefit from state support and alter their long-term investment strategies to become more exposed to riskier sectors in host countries in exchange for higher returns or greater influence. There might be some interesting coordination among firms in the same industry through investments by sovereign wealth funds and state-owned firms that reinforce state influence in that industry, not only at home but also abroad.

#### Influence

Research can analyze whether and how corporate governance standards at the holding or portfolio levels diffuse to the next micro-level of the subsidiary or investee firms in host countries. Sovereign wealth funds could potentially become better monitors among institutional investors since they have long-term commitments in their investee firms and lack liquidity constraints. However, given the political and social priorities of sovereign wealth fund board representatives, it is unclear what the overall impact on the corporate governance processes of their investee firms would be, creating an opportunity for further research. We need more research on the management of foreign subsidiaries of state-owned multinationals and foreign portfolio firms of sovereign wealth funds, such as the acquisition and transfer of knowledge from host to home operations. There is also the issue of heterogeneity in the level of ownership in foreign investments and the varying degrees of government influence. Minority ownership, for example, is often considered a subtle but still influential method of government intervention (Inoue et al., 2013).

#### Impact

Another area of interest is how host countries manage the trade-offs between the economic benefits and political costs of investments by state-owned multinationals and sovereign wealth funds. There is a conflict between the technocratic analysis of the economic benefits of investments by state-owned multinationals and sovereign wealth funds, and the politically driven discourse about the interference of home governments in host-country economies. State-owned multinationals can generate spillovers from investing in knowledge transfer and knowledge-intensive industries, such as high tech, and be proactive in such spillovers, instead of aiming to reduce them, as is the usual recommendation of the literature (Blomström & Kokko, 1998). Investments by state-owned multinationals and sovereign wealth funds can be used as rewards or punishments, but there is a need for a more subtle understanding of the differences between state-owned multinationals and sovereign wealth funds as tools of foreign influence and power. Sovereign wealth funds usually have fungible investments that can be more easily changed, but are less influential because of their smaller size. In contrast, state-owned firms have large stakes that grant firms greater influence, but reduce their ability to exit the host country quickly.

### Home- and Host-Country Relationships: Conflicts, Mediation, and Disguising

The impact of the interactions between the host and the home country on the behavior of state-owned multinationals and sovereign wealth funds is a topic with ample potential for future research. The typical approach to the analysis of home and host connections is to identify the differences between home- and host-country settings and to study how these reduce international expansion and success (Berry, Guillén, & Zhou, 2010; Beugelsdijk, Kostova, Kunst, Spadafora, & van Essen, 2018). The study of state-owned entities goes beyond these differences and into the quality of the relationships between countries, because what matters for state-owned entities is the relationship between home- and host-country governments.

#### Conflicts

There are intriguing instances of state-owned entities addressing political conflicts between home and host countries. It would be interesting to investigate the institutional variety of responses to foreign government investments. For instance, some EU countries have proactively sought investments from sovereign wealth funds of countries with which they had strong political and trade links (Thatcher & Vlandas, 2016). It is interesting to analyze host-country retaliation, that is, how countries can use evaluations and other informal political restrictions to limit hostile governments’ foreign investments (Cuervo-Cazurra, 2018; Lavelle, 2017). For example, the Committee on Foreign Investment in the United States limits foreign governments’ influence through state-owned multinationals and sovereign wealth funds.

#### Mediation

However, government-owned entities do not have to be passive captives in conflicts between home and host governments. Instead, they could mediate relationships through informal channels to achieve their goals. Internationalization and financialization imply that financial markets contain power projection mechanisms, which governments can deploy via foreign investments of state-owned firms or sovereign wealth funds (Monk, 2011).

#### Disguising

In addition to host-country governments, state-owned entities need to pay attention to the perceptions of host-country citizens. Host governments and citizens react differently to entry by state-owned entities. Whereas governments are aware of and have national security concerns about investment by foreign sovereign wealth funds or state-owned multinationals (Cuervo-Cazurra, 2018), citizens are often unaware of the state ownership of firms. Government-owned entities can respond to these reactions by disguising their influence. For example, state-owned multinationals may retain the brand names of local companies and products they purchase to disguise their foreignness, while sovereign wealth funds can use private equity funds to invest abroad indirectly.

### Management: Orientation, Opacity, and Arbitrage

As to the management of state-owned entities, it would be interesting to understand how they are managed to balance the need to achieve business objectives and the requirement of politicians to pursue some nonbusiness objectives. A few critical issues worth considering are manager orientation, decision opacity, and investment arbitrage.

#### Orientation

Research on managerial behavior could illuminate the decision-making processes within state-owned multinationals’ subsidiaries and sovereign wealth funds’ investee firms. For example, future research could investigate whether the long-term orientation of the government impacts the strategic decision-making of managers in subsidiaries/investee firms in host countries. Additionally, future studies could focus on the effect of sovereign wealth fund shareholder activism on managerial behavior in investee firms (Wright & Amess, 2017). Also important to study is investment-level heterogeneity in areas such as size, fit with the portfolio or holdings, and other influences on managerial attention and internationalization.

#### Opacity

One opportunity would be to study the determinants of sovereign wealth fund transparency and disclosure to find out why some sovereign wealth funds choose to remain opaque despite restrictions when investing abroad. Considering their opacity as a facilitator of money laundering and embezzlement, research could investigate the mechanisms that mitigate expropriation, especially since sovereign wealth funds are increasingly intertwined with the global financial markets through their foreign investments.

#### Arbitrage

Finally, further research is needed on the heterogeneity of state-owned multinational and sovereign wealth fund investments. Namely, research on the varying role of the state as a shareholder in state-owned multinationals relative to privately held multinationals in the valuation effects can be useful in understanding the motives behind international investments. Prior studies have shown that international corporate diversification enhances firm value in private multinationals (Errunza & Senbet, 1981; Gande, Schenzler, & Senbet, 2009), but further light can be shed on the effect of international diversification in state-owned vehicles, and how the latter arbitrage differences in legal forms of incorporation and corporate taxes across countries relative to private multinationals (Gande, John, Nair, & Senbet, 2020).

## Extending Theories of the Firm via Cross-Fertilization

Complementing the analysis of new research avenues on the internationalization of state-owned multinationals and sovereign wealth funds discussed in the previous section, we suggest another extension by refining theoretical explanations via cross-fertilization. We illustrate this by explaining how selected theories of the firm – agency, transaction cost economics, the resource-based view, resource dependence, and neo-institutional – can be advanced via the cross-fertilization of insights from one research area to the other. Table 8 summarizes these ideas.

**Table 8:** Theoretical bridges between state-owned multinationals and sovereign wealth funds

	Insights from state-owned multinationals	Insights from sovereign wealth funds	Cross-fertilization from state-owned multinationals to sovereign wealth funds	Cross-fertilization from sovereign wealth funds to state-owned multinationals
Agency	State-owned multinationals suffer from multilevel principal-agent problems among citizens, politicians, and managers, with an additional principal-principal conflict for minority state-owned firms that reduce their competitiveness and thus limit internationalization. The internationalization of state-owned firms implies that management of international and distant investments is delegated down the line to the manager of that investee firm or subsidiary	Sovereign wealth funds suffer from a multi-principal agency among the government and private investors in target firms that create conflicts in the invested firms. The autonomy and attention of sovereign wealth funds’ managers differ depending on the size and type of investment	Analyze sovereign wealth funds’ multilevel problems among their managers, citizens, and politicians, and managers of sovereign wealth fund-invested firms	Analyze state-owned multinationals’ multi-principal conflicts in partially state-owned firms and subsidiaries abroad. Analyze investment decisions by state-owned multinationals involving indirect stakes in complex ownership pyramids.
Transaction cost economics	State-owned multinationals’ cost of transacting is decreased due to better control by the government of fraudulent behavior within state-owned multinationals, and that facilitates their ability to transact with governments in other countries	Sovereign wealth funds can deal with higher levels of risks due to the support of the government, target firms and recipient countries benefit from lower transaction costs, patient capital, and access to sovereign wealth funds’ transacting markets	Analyze sovereign wealth funds’ transaction costs savings from investing in countries with high government intervention	Analyze how the costs of transacting for state-owned multinationals in host countries is reduced from access to state-owned multinationals' home countries
Resource-based view	State-owned multinationals benefit from more accessible access to state resources and use this to support their foreign expansion	Sovereign wealth funds help reduce the resource curse in the country because they diversify risk in global investments	Analyze sovereign wealth funds’ access to state resources advantage effect on the selection of investments	Analyze state-owned multinationals’ ability to reduce the resource needs of the home country via their foreign investments
Resource dependence	State-owned multinationals depend on the state for support and decision making that limits their ability to internationalize	Sovereign wealth funds decrease dependence and political interference by the government via foreign investments	Analyze sovereign wealth funds’ increased dependence on government support in foreign investments	Analyze state-owned multinationals decreased political interference in their foreign investments
Neo-institutional	State-owned multinationals suffer from a lower legitimacy in host countries that limits their ability to control firms abroad. Home-country institutions shape the ability of stakeholders to monitor and influence the internationalization of state-owned multinationals. Minority state ownership adds a layer of complexity to the institutional moderation of the ownership–internationalization relationship	Sovereign wealth funds are used as tools to influence the legitimacy of the home country abroad, or as tools of their home-country geopolitical and national economic development	Analyze sovereign wealth funds’ lower legitimacy across host countries that limits the size of their investments. Analyze institutional diversity established through the varieties of capitalism, and how it moderates the ownership–internationalization relationship in sovereign wealth funds	Analyze state-owned multinationals’ use as tools to change the legitimacy of the government, or as enablers of national economic development

### Agency Theory

Agency theory is primarily concerned with how to align the interests of principals (owners) and agents (managers) to achieve desired goals (Jensen & Meckling, 1976). State-owned firms suffer from unique agency problems in comparison to private firms. These firms are viewed as suffering from multilevel principal-agent problems which result in reduced competitiveness and performance relative to private firms (Grøgaard et al., 2019; Grosman et al., 2019; Musacchio & Lazzarini, 2014). These conflicts among the goals of citizens (the nominal owners and ultimate principals), politicians (the agents of citizens), civil servants (the agents of politicians), and managers (the agents of politicians and civil servants) depend on the organizational distance between principals and agents. Some state-owned multinationals are directly influenced by their governments, while others, especially minority-owned ones, are more autonomous (Inoue et al., 2013). An interesting theoretical dilemma for state-owned firms is that greater managerial autonomy increases agency conflicts, but at the same time it reduces political interference in decisions. These insights can help extend research on sovereign wealth funds by discussing how divergence in objectives among citizens, politicians, and asset managers influence the behavior of sovereign wealth funds and their investee firms abroad. Another extension is analyzing state-ownership opacity and the expropriation of minority shareholders (Grosman et al., 2019; Sun, Hu, & Hillman, 2016). Research on sovereign wealth funds can benefit from these insights, given that their opacity makes it easier to misappropriate funds (Rose, 2017).

Sovereign wealth fund research can help inform new agency approaches in state-owned multinationals. Agency problems are exacerbated when sovereign wealth funds invest indirectly through intermediaries like private equity or hedge funds (Wright & Amess, 2017). Research on state-owned multinationals can incorporate more detailed analyses of multi-principal conflicts in firms and foreign subsidiaries with mixed ownership. Additionally, the autonomy and attention of sovereign wealth funds’ managers vary with the size and type of investment (Makhoul et al., 2020), which can inform studies of state-owned multinationals and their stakes in complex ownership pyramids.

### Transaction Costs Economics

Transaction costs economics explains firm behavior based on reducing the cost of transactions in economic relationships among actors (Williamson, 1985). State ownership has competing influences on transaction cost management. The negative view of government ownership argues that it increases the transaction costs by augmenting the risk that a firm may not fulfill contract obligations due to politically motivated interference. The positive view argues that it decreases transaction costs by reducing the risk of fraudulent behavior on behalf of firms. Which effect prevails depends on contingencies. For instance, state-owned multinationals are better able to manage global risks and institutional challenges from their experience in dealing with the uncertainty and risk of transacting with a government. This can be extended to the sovereign wealth fund literature to analyze how this experience helps them make better investments in countries with high government intervention.

The literature on sovereign wealth funds uses transaction costs economics differently, proposing that sovereign wealth funds can deal with higher levels of investment risk due to the government’s support. Recipient target firms and countries benefit from access to patient (long-term) capital and sovereign wealth fund home-country markets (Rose, 2009). The state-owned multinationals literature can benefit by incorporating these ideas to explain how the support of the home government helps state-owned multinationals reduce their transaction costs when investing in locations with a higher risk profile.

### Resource-Based View

The resource-based view explains how private firms develop and use resources and capabilities to satisfy customers’ needs better than rivals (Barney, 1991). From this viewpoint, state ownership provides an advantage from access to privileged funding, regulation, and support, thanks to the human and social capital of government officials appointed to the boards that also facilitates internationalization. Studies on sovereign wealth funds can use these ideas to explain how sovereign wealth funds gain an advantage from access to state resources, not only as capital but also from connections to government officials and their expertise in dealing with foreign investments and governments.

The literature on sovereign wealth funds does not explicitly build on the resource-based view. Instead, it discusses how sovereign wealth funds mitigate the resource curse by reducing the negative consequences of natural resource wealth, reducing corruption from the misuse of wealth in emerging economies (Dixon & Monk, 2011). Alternatively, sovereign wealth funds help home governments access resources abroad to promote national development (Haberly, 2011; Kamiński, 2017; Sun et al., 2014). Studies of the building of state-owned multinationals can be cross-fertilized with these ideas to analyze their ability to reduce the resource needs of the home country via their foreign investments.

### Resource Dependence Theory

Resource dependence analyzes power, which is driven by one actor influencing the behavior of another because the former controls something the latter needs (Pfeffer & Salancik, 1978). State-owned firms depend on the government for resources and guidance, constraining their ability to pursue business objectives. This also limits their ability to invest abroad, as the dependence enables politicians to induce state-owned multinationals to invest at home (Deng et al., 2018; Okhmatovskiy, 2010). This sometimes leads managers of state-owned firms to try to escape politicians’ control via internationalization (Choudhury & Khanna, 2014; Rodrigues & Dieleman, 2018). These ideas can inform the study of the constraining effects of government influence on sovereign wealth funds’ capital allocation strategies (Vasudeva, 2013) and how politicians’ preferences drive their investments.

Studies on sovereign wealth funds do not explicitly build on a resource dependence approach, but they point out the challenges that some economies and sovereign wealth funds encounter from their dependence on natural resources as their primary sources of income in their asset allocation strategies (Balding & Yao, 2011). Such dependence induces sovereign wealth funds to invest in a broader range of industries abroad to lessen dependence on the home government regulatory framework (Megginson & Fotak, 2015). State-owned multinationals research can use these insights to study how foreign expansion into diverse industries can reduce dependence on home-country institutions (Choudhury & Khanna, 2014).

### Institutional Theory

Neo-institutional theory proposes that, to achieve legitimacy, firms imitate other companies in response to host-country regulatory, normative, and cognitive pressures. In the context of state-owned firms, it notes that these firms suffer from state ownership illegitimacy abroad. The effect of state ownership on internationalization switches from being negative in liberal market economies to positive in coordinated market economies at either extreme of the varieties of capitalism spectrum (Mariotti & Marzano, 2019). Comparisons between minority and majority state ownership add another layer of complexity to the institutional moderation (Benito et al., 2016; Chen et al., 2019; Grøgaard et al., 2019; Mariotti & Marzano, 2019). Research on sovereign wealth funds can study how their lower legitimacy abroad limits their investments, the ownership of noncontrolling positions, and investments under the disclosure thresholds in the host country.

Sovereign wealth funds are efficient and powerful tools for the home governments to indirectly influence the legitimacy and perception of a country in which they invest (Vasudeva et al., 2018). There is always a fear that, behind the seemingly rational financial investments, there is a political or social agenda, even if it promotes democratic values such as national welfare or global justice (Dixon & Monk, 2012; Monk, 2011). These ideas can cross-fertilize research on state-owned multinationals by analyzing the political influence through their investments abroad, discussing how the political agenda of the home government modifies the perceptions of legitimacy.

## Emerging tTheoretical Construct: Discreet Power

Finally, we complement the review and guidance on future research topics and theories with an additional suggestion for theoretical advancement by introducing the concept of discreet power. This concept encapsulates discussions in previous studies of the use of state-owned multinationals and sovereign wealth funds or both as vehicles for influencing host-country governments. This is a suggestion for clarifying past insights and providing additional theoretical depth, which future research can refine.

### Power in International Relations: Hard, Soft, and Discreet

Power is the ability to influence others to follow one’s desires. It is a relative concept in which one party can induce another to change its behavior according to the first party’s preferences. In relations between countries, the traditional typology of power is the distinction between hard and soft power (Nye, 2004). On the one hand, hard power is associated with one government influencing another through coercion. The latter acquiesces because it fears the potential retaliation in case of non-compliance. Hard power is usually tied to military and economic might (Robertson & Sin, 2017). The retaliation threat can come from using military force and armed conflict to force alignment. It can also be driven by economic strength and the threat of loss of economic relations in cases of misalignment of behavior (Layne, 1997).

On the other hand, soft power is linked to one country influencing the behavior of another through co-optation, with the latter being attracted to the former and adopting the desired behavior voluntarily (Nye, 2004). Co-optation can result from policies that the country adopts in its international relations and that benefit other countries, leading to their adoption. It can also be motivated by the political values espoused by the country through its foreign policy, which becomes a reference that other countries see as legitimate and emulate (Gill & Huang, 2006). Finally, it can be led by the country’s culture and values, which are perceived to be superior by others and are thus imitated (Watanabe & McConnell, 2008).

We suggest discreet power as a third option. With discreet power, the government aims to provide a subtle inducement for aligning behavior through nudging. A nudge is “any aspect of the choice architecture that alters people’s behavior […] without […] significantly changing their economic incentives” (Thaler & Sunstein, 2008: p.6). Governments can nudge other countries toward their objectives with investments in the host country that follow the preferences of the government, with such investments being undertaken by state-owned companies or financed by state-owned banks and sovereign wealth funds. Thus, discreet power falls in between hard and soft power. It is neither the result of the first country forcing the second to adopt policies nor the second adopting the policies voluntarily, but rather part of a more subtle mutually beneficial relationship between the two countries. One example of discreet power via sovereign wealth funds is the global diffusion of values espoused by the Norwegian government through its use of Norway’s Government Pension Fund Global (Rose, 2017; Vasudeva, 2013) by engaging in shareholder activism.

### State-Owned Multinationals and Sovereign Wealth Funds as Tools of Discreet Power

State-owned multinationals and sovereign wealth funds can be used as mechanisms for achieving discreet power in two ways: as a *reward* that highlights the host-country’s benefit for aligning interest, or as a *punishment* that points to the harm to the host country for not following expected behavior. On the one hand, the home government can follow a reward approach in its use of discreet power via state-owned multinationals and sovereign wealth funds to nudge its political priorities abroad. The home government can guide its state-owned firms to build subsidized infrastructure in the host country, such as roads, railroads, or electricity distribution (Mwase, 1983). These investments are highly visible to local politicians and populations and help improve relations with the host country, aligning interests in bilateral cooperation and gaining support in multilateral institutions (Dreger, Schüler-Zhou, & Schüller, 2017). The home-country government can use the sovereign wealth funds to invest in preferred projects in the host country, so that these projects have the funding needed to become viable when commercial providers of funds would shy away from the costs or risks (Haberly, 2011). Such investments can help improve political relationships with the host government and keep political preferences aligned (Beeson, 2009). The reward approach is thus likely to be more efficient when there are affinities between the governments of home and host countries that foster existing political or trade relations further.

On the other hand, the home government can use punishment through discreet power tactics via its state-owned multinationals and sovereign wealth funds when political interests are not aligned. It can direct its state-owned firms to limit investments or reduce the purchases of imports from the host country (Yang, Zhang, & Terazono, 2019), as was done, for example, when in 2021 China punished Australia’s attempt to investigate how the COVID-19 virus emerged. Such actions can be a subtle message to the host country to nudge its political actions to align with the desires of the home government, given that a reduction in the purchase of imports has a quick and reversible impact. The government can also direct its sovereign wealth funds to divest from host-country firms to nudge the host-country government to align with political desires, as the opening quote illustrated. Such divestitures have expediency value, since the sale of stocks and bonds can be quickly reversed once the preferred political behavior is adopted.

### Discreet Power Strategies across Countries

All countries can use state-owned multinationals and sovereign wealth funds as mechanisms to implement discreet power, but their use is likely to vary across countries. We propose four types of nonbusiness strategies of state-owned entities as tools of discreet power: recognition, values, development, and supremacy. Table 9 illustrates the classification of discreet power strategies by whether they are used with a mostly social or economic goal, and whether the orientation is mostly inward or outward. These nonbusiness strategies complement the business strategies of the state-owned entities and their search for financial returns. The discreet power strategies evolve with changes in the political regime, ideology, or leadership. For example, under Prime Minister Justin Trudeau, the government of Canada used its sovereign wealth fund to promote the sale of oil and increase economic returns, rather than spread its social and ethical values to other countries, as was more the case previously. Nevertheless, they are likely to be used more or less intensively depending on the characteristics and attitudes of governments.

**Table 9:** Types of strategies of state-owned multinationals and sovereign wealth funds to achieve discreet power

		Orientation	
		Inward	Outward
Goal	Social	**Recognition** The government uses state-owned multinationals and sovereign wealth funds to improve the country’s recognition by other countries	**Values** The government uses state-owned multinationals and sovereign wealth funds to spread its ethical and social values to other countries
	Economic	**Development** The government uses state-owned multinationals and sovereign wealth funds to facilitate the country’s development	**Supremacy** The government uses state-owned multinationals and sovereign wealth funds to achieve supremacy in other countries

A *value strategy* is the use of discreet power to spread home values in other countries. It is likely to be used by countries with democratic governments in market-oriented states. Strong institutions and the separation among powers in democratic systems limit the ability of politicians to force state-owned firms towards nonbusiness objectives. Thus, state-owned firms are likely to internationalize in pursuit of business objectives, as in the case with German automobile firm Volkswagen’s global expansion, while sovereign wealth funds invest in pursuit of financial returns, as in the case of the Alaska Permanent Fund Corporation’s mandate to obtain sustained, compelling returns for Alaskans. Their internationalization may be influenced by the desire to spread preferred values and ethical standards abroad, as occurred when Norway’s Government Pension Fund Global became an instrument of the Norwegian government for spreading social norms and environmental practices (Vasudeva, 2013). This strategy is likely to be most effective when the sovereign wealth fund coordinates with other investors to alter company values. The value strategy is most likely to be deployed through sovereign wealth funds, since most state assets in such countries are owned through them, although welfare states also deploy the value strategy through state-owned firms, as with Italian ENEL’s push to develop sustainable power grids in emerging countries.

A *supremacy strategy* is the utilization of discreet power to exert dominance abroad. It is more likely to be followed by interventionist countries with authoritarian regimes, where the dominant channel for discreet power is through state-owned firms. These countries have governments with limited checks and balances on the use of state-owned firms by politicians, who can induce state-owned firms to undertake the investments and activities that politicians deem useful for them. Politicians can direct state-owned firms to invest in preferred countries to achieve political goals, as when Russia’s state-owned Gazprom invested in developing a pipeline to Germany that bypasses Ukraine. They can also use sovereign wealth fund investments to support political relationships despite dubious financial returns, as when Libya’s sovereign wealth fund bought 25% of a $4 billion issuance of convertible bonds by Unicredit, Italy’s second-largest bank, and later became the largest shareholder (Bertoni & Lugo, 2013).

A *development strategy* is the use of discreet power in host countries to facilitate the home-country’s development. It is likely to be employed by interventionist states with democratic governments, and primarily through state-owned firms. The checks and balances of the democratic government limit the use of state-owned firms to exert political influence abroad if this results in a misuse of funds. Politicians and civil servants can guide state-owned firms or funds to invest in international projects that facilitate access to needed technologies or markets, such as the Indian government mandating mineral-based state-owned firms to buy strategic lithium and cobalt assets overseas, as India aims to build an electric vehicle battery manufacturing capacity (Majumdar, 2018).

Finally, the *recognition strategy* is using discreet power to improve the home-country’s standing abroad. It may be employed by entrepreneurial states with authoritarian political regimes, primarily through sovereign wealth funds. In these countries, the authoritarian nature of the governments reduces criticism. Political objectives can take preeminence over commercial ones in foreign investments as the government creates firms that strengthen its recognition abroad. An example is the sovereign wealth fund Investment Corporation of Dubai, which assumed the costs of hedging oil contracts for the United Arab Emirates’ airline Emirates to help its growth, increasing Dubai’s recognition in the region (Saleem, 2015).

## Conclusions

The literature on state capitalism in international business has been ramping up in recent times, highlighting the importance of the phenomenon whereby governments seek ownership or control of foreign assets. However, separate theories of multinationals and institutional investors have been developed to explain the behavior of private firms. Hence, we need an extension to explain governments as foreign investors and to describe how their nonbusiness objectives affect the internationalization of state-owned multinationals and sovereign wealth funds. This review explains how the government imposes nonbusiness objectives that alter the internationalization of state-owned firms and sovereign wealth funds, opening new topics and theorization opportunities. We outline suggestions for extending the literature in topics, theoretical cross-fertilization, and the concept of discreet power as a theoretical construct that encapsulates previous ideas.

### Contributions to the Literature

This article makes several significant contributions to our understanding of the role of the government as a foreign investor. The review clarifies the state-of-the-art on the topic, promoting the need to establish bridges between the study of the internationalization of sovereign wealth funds and state-owned firms and paying attention to the role of governments’ nonbusiness objectives on foreign investments. We complement the independent reviews on sovereign wealth funds in the economics and finance literature (Bahoo et al., 2020; Megginson & Fotak, 2015; Megginson & Gao, 2020) and on state-owned multinationals in international business (Cuervo-Cazurra & Li, 2021; Cuervo-Cazurra et al., 2014) by clarifying how insights in each stream can help promote a better understanding of the other.

The review highlights the importance of governments’ nonbusiness drivers of internationalization and their strategic implications. Foreign investments by governments through state-owned multinationals and sovereign wealth funds are subject to a balance between their business objectives as economic agents and their nonbusiness objectives as state-owned entities. This balancing results in a much more subtle impact of government ownership than the traditional negative view. Instead, there is a duality in state ownership, as it can harm some internationalization decisions while helping others. This duality view can help future researchers develop more sophisticated predictions by analyzing the conditions under which the helping hand may compensate the grabbing hand, and vice versa, not only in internationalization but also in other strategic decisions such as growth, diversification, or performance.

The joint study of state-owned multinationals and sovereign wealth funds helps advance theory in several ways. We suggest expanding theories of the firm by cross-fertilizing insights from one topic on the other. Additionally, we suggest the concept of discreet power to clarify the understanding of the government as a foreign investor. This is a new dimension of power that complements the traditional binary view of hard and soft power in international relations (Nye, 2004). The notion of discreet power highlights the use of nudging as the mechanism by which governments can align the interests of other countries to their own, be it of a rewarding or a punishing nature. Our concept of discreet power is further calibrated by providing a typology of four strategies for using it based on whether its orientation is inward or outward and whether the type of desired goals is economic or social. Our categorization of uses of discreet power implicitly builds on the law and finance literature (Djankov, La Porta, Lopez-de-Silanes, & Shleifer, 2002) by bringing in the importance of politics, laws, and regulations on strategy. A better understanding of the discreet power of governments could further elucidate the institutional varieties of these economies. Further, the intensity of discreet power can be tested depending on the level of activity in foreign policy (Lavelle, 2017). The study of discreet power can be extended to investments by private firms with the government supporting private national champions, with the view that some foreign ventures can contribute to improving relations and aligning interest with host-country governments. The literature on politically connected private companies usually considers firms using the government to achieve their objectives (Faccio, 2006), and can also be extended by considering discreet power strategies. In sum, we offer a complementary view of the government exercising discreet power through state-owned entities in a symbiotic relationship.

### Contributions to Practice

Our conceptual framework can help decision-makers better understand the role of governments in international business. There is much debate on the wisdom of opening to investments by state-owned multinationals and sovereign wealth funds with the view that the governments behind them might use these firms to pursue their nonbusiness agendas (Cuervo-Cazurra, 2018). We clarify when this might be the case and discuss the logic and mechanisms that enable their use to influence local governments. This does not mean that host-country governments should block all investments by state-owned multinationals and sovereign wealth funds. On the contrary, these investors can bring much-needed technological and financial support for the firms in which they invest. The host country can analyze the strategy of discreet power behind the investment and adopt appropriate responses. Whereas governments from autocratic and institutionally weak economies might use state-owned multinationals and sovereign wealth funds to exert undue influence in host countries, those from democratic and institutionally strong economies can be used to bring superior governance and ethical standards.

## Supplementary Information

Below is the link to the electronic supplementary material.

Supplementary file 1 (DOCX 420 kb)
