# Supplementary material for: A review of the internationalization of state-owned firms and sovereign wealth funds: Governments’ nonbusiness objectives and discreet power
Source: J Int Bus Stud. 2022 May 11;54(1):78–106. doi: 10.1057/s41267-022-00522-w (PMC9090597; doi:10.1057/s41267-022-00522-w)
Supplement: Supplementary file 1 — (DOCX 420 kb) [file 41267_2022_522_MOESM1_ESM.docx]

**ONLINE APPENDIX TO ACCOMPANY**

**A REVIEW OF THE INTERNATIONALIZATION OF STATE-OWNED FIRMS AND SOVEREIGN WEALTH FUNDS: GOVERNMENTS’ NONBUSINESS OBJECTIVES AND DISCREET POWER**

Alvaro CUERVO-CAZURRA

Northeastern University, D’Amore-McKim School of Business

360 Huntington Avenue, 313 Hayden Hall, Boston, MA 02115-5000, USA

Phone: +1-617-373-6568, email: a.cuervocazurra@neu.edu

Anna GROSMAN

Loughborough University London

3 Lesney Avenue, Here East, Queen Elizabeth Olympic Park, London E20 3BS, UK

Phone: +44 203 805 1307, email: a.grosman@lboro.ac.uk

William L. MEGGINSON

The University of Oklahoma, Michael F. Price College of Business

307 W. Brooks, Suite 205B, Norman, OK 73019, USA

Tel.: +1 (405) 325-2058. e-mail: wmegginson@ou.edu

Visiting Professor, University of International Business & Economics (Beijing)

December 15, 2021

Review Article for *Journal of International Business Studies*

**RESEARCH DESIGN AND PAPERS ANALYZED**

We perform a content analysis of past literature on the internationalization of state-owned firms and sovereign wealth funds to better understand the current conceptualization of the government as a foreign investor. Content analysis is a technique that enables us to identify the argument of studies regardless of the methodology they used, and thus we review theoretical, qualitative, and quantitative articles (Duriau, Reger, & Pfarrer, 2007; Gaur & Kumar, 2018). We do not do a meta-analysis because this technique is restricted to quantitative research (Geyskens, Krishnan, Steenkamp, & Cunha, 2009; Lipsey & Wilson, 2001).

Mapping the connections among these two streams of literature reveals a significant opportunity to establish bridges that past studies seem to have overlooked. We use the VOSviewer tool (VOSViewer, 2019) to visualize relationships among academic articles. We first run a search in Web of Science of peer-reviewed articles that have the keywords “state-owned multinational” and “internationalization” or “sovereign wealth fund” and “internationalization” in the abstract. This search yields 260 articles. We then create a map based on bibliographical data with the ris format, using the co-occurrence and full counting method and selecting a minimum of five occurrences of a keyword. Figure A provides the relationships among topics. We find a large cluster around government business enterprise and its connections to foreign investments, internationalization, and globalization, a secondary cluster that connects state-owned enterprises, international business enterprise, and emerging markets, and a third smaller cluster on sovereign wealth funds and business enterprises.

Figure A. Connections among the streams of literature analyzing state-owned multinationals and sovereign wealth funds


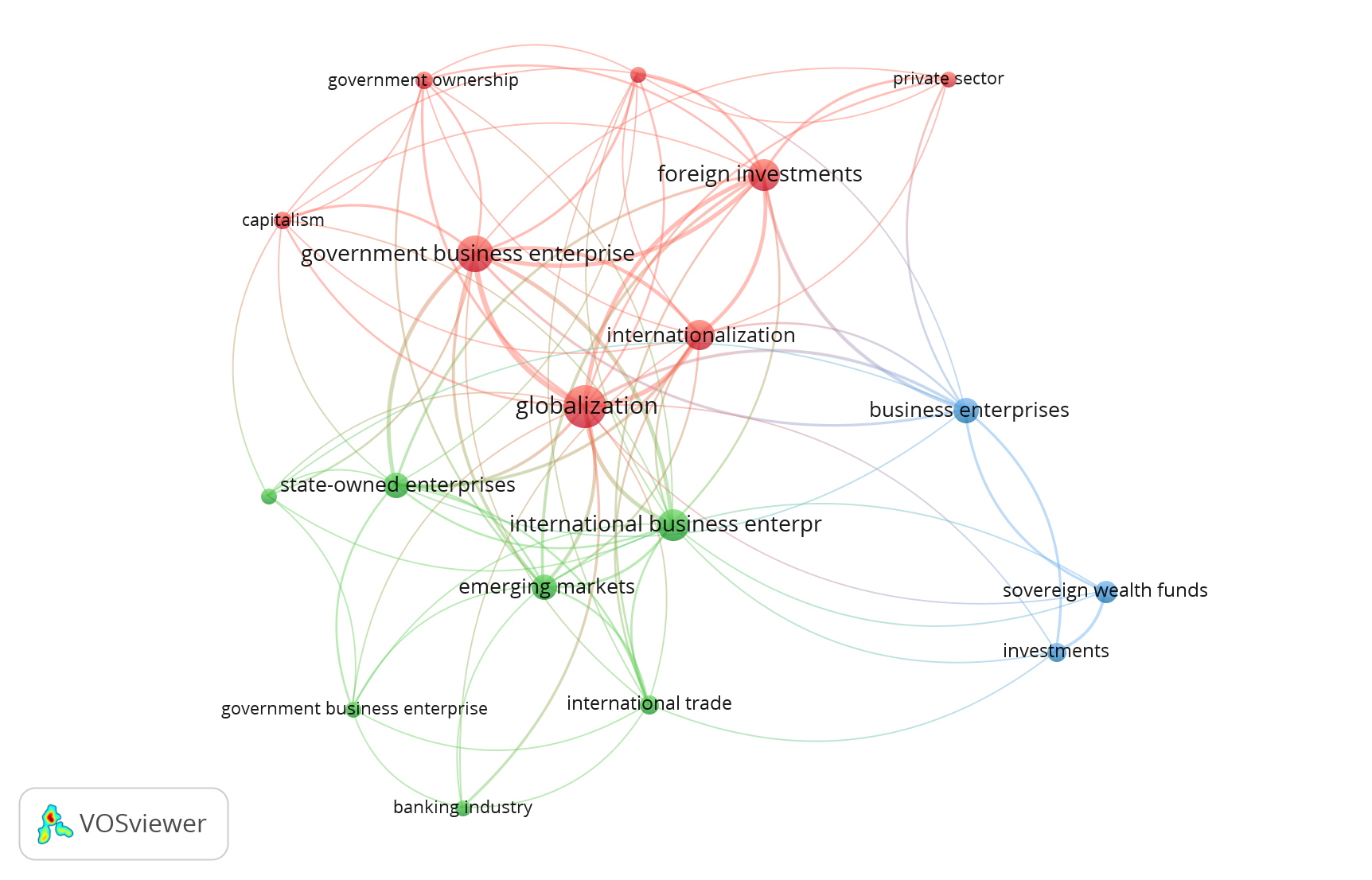


Note: The distance between two items in the visualization approximately indicates the relatedness of the items in terms of co-citation links. In general, the closer two items are located to each other, the stronger their relatedness. The strongest co-citation links between items are also represented by lines. There are three clusters: a red cluster around government business enterprise and its connections to foreign investments, internationalization, and globalization, a green cluster that connects state-owned enterprises, international business enterprise, and emerging markets, and a third smaller cluster in blue on sovereign wealth funds and business enterprises.

Following the recommendations of previous content analysis research (Canabal & White III, 2008; Terjesen, Hessels, & Li, 2016), we use five steps. In the first step we select journal articles published in English that appeared in scholarly journals listed in EBSCO and that contained the keywords ‘state-owned’ & ‘multinational’, ‘state-owned multinational’, ‘state-owned’ & ‘foreign investment’, ‘state-owned’ & ‘foreign direct investment’, ‘state-owned’ & ‘foreign portfolio investment’, ‘state-owned’ & ‘cross-border acquisitions’, ‘state-owned’ & ‘international alliance’, ‘state-owned’ & ‘international joint ventures’, ‘state-owned’ & ‘internationalization’, ‘government-linked’ & ‘multinational’, ‘government-linked multinational’, ‘government-linked’ & ‘foreign direct investment’, ‘government-linked’ & ‘foreign portfolio investment’, ‘government-linked’ & ‘cross-border acquisitions’, ‘government-linked’ & ‘international alliance’, ‘government-linked’ & ‘international joint ventures’, ‘government-linked’ & ‘internationalization’, ‘sovereign wealth fund’ & ‘foreign investment’, ‘sovereign wealth fund’ & ‘internationalization’, ‘sovereign wealth fund’ & ‘investment abroad’, ‘state pension fund’ & ‘foreign investment’, ‘state pension fund’ & ‘internationalization’, ‘state pension fund’ & ‘international’, or ‘state pension fund’ & ‘investment abroad’.

In the second step, we restrict articles to those published in top journals in management, economics, economic geography, finance, and international business. We consider top journals as those in the Financial Times Top 50 journals list (Ormans, 2016): *Academy of Management Journal* (AMJ), *Academy of Management Review* (AMR), *Administrative Science Quarterly* (ASQ), *American Economic Review* (AER), *Econometrica* (E), *Journal of Finance* (JF), *Journal of Financial and Quantitative Analysis* (JFQA), *Journal of Financial Economics* (JFE), *Journal of International Business Studies* (JIBS), *Journal of Management* (JOM), *Journal of Management Studies* (JMS), *Journal of Political Economy* (JPE), *Management Science* (MS), *Quarterly Journal of Economics* (QJE), *Organization Science* (OS), *Organization Studies* (OrgS), *Review of Economic Studies* (RES), *Review of Finance* (RF), *Review of Financial Studies* (RFS), and *Strategic Management Journal* (SMJ). We complement this list with other top journals in management, economics, economic geography, political economy, finance, and international business, considering those ranked as three or above in the list of the Association of Business Schools (CABS, 2019) as done previously (Gaur & Kumar, 2018; Tüselmann, McDonald, & Thorpe, 2006).

In the third step, we read the titles and abstracts of all articles, and if the article appears to analyze the internationalization of state-owned firms or sovereign wealth funds, we study its content to make sure that it focuses on our topic of interest. From this step, we end up with 73 articles analyzing the internationalization of state-owned firms and 26 on the internationalization of sovereign wealth funds.

In the fourth step, we analyze the content of the articles and systematically summarize their ideas to facilitate the identification of insights and the comparison of the arguments and conclusions. We organize the summary under the following categories: article (author, year, publication), research question; sample (if quantitative); and main arguments and findings. Tables A and B summarize the papers analyzed on the internationalization of state-owned multinationals and sovereign wealth funds, respectively.

In the fifth step, we review other relevant articles, book chapters, and books and build on their ideas when appropriate. However, we do not include them in the summary tables since they were not published in the journals included in our research design.

Table A. Summary of selected studies on the internationalization of state-owned multinationals

| **Author (Year)** | **Journal** | **Research Question** | **Sample** | **Main Arguments and Findings** |
| --- | --- | --- | --- | --- |
| Franko (1975) | JIBS | To what degree policies of home governments, rather than markets, condition internationalization of state-owned enterprises | Not applicable | State-owned enterprises internationalize to pursue industrial policy goals such as securing natural resource access. |
| Mazzolini (1979) | JIBS | Explain the behavior of European government-controlled enterprises | 123 state-owned enterprises in nine European countries in 1975-1979 | State-owned enterprises are constrained in focusing at home, going abroad to obtain resources, support the currency. Foreign investments require approval and are bureaucratic. |
| Vernon (1979) | JIBS | Identify the practices of state-owned enterprises | Not applicable | State-owned enterprises are created as national champions, fiscal agents, monopolists, industrialization promoters, bilateral trades, which creates a multiplicity of goals, leading to the use of domestic inputs, exports, and support other state-owned enterprises, leading to lower international competitiveness. |
| Zutshi and Gibbons (1998) | APJM | The internationalization process of state-owned enterprises in Singapore | Two government-linked companies in Singapore | Internationalization is a direct response to the government’s policy. The state-owned enterprises are direct arms and modalities for implementing such government policies. |
| Aharoni (1980) | JWB | State-owned enterprises as competitors in the international market | Not applicable | New competitors that deserve more attention. |
| Mazzolini (1980) | JIBS | Analyze the leadership influence of state-owned enterprises in their internationalization | 124 state-owned enterprises in nine European countries in 1975-1979 | Politicians are short-term oriented, seek visible results, and take partisan views, leading to less internationalization, more employment, and unclear decisions. |
| Mazzolini (1980) | SMJ | Why state-owned enterprises internationalize and what is special about their internationalization | 125 state-owned enterprises in nine European countries in 1975-1979 | State ownership reduces internationalization as information processed and bureaucracy are focused on home, with politics further constraining internationalization. |
| Rugman (1983) | MIR | What are the differences in the performance of US and European multinationals | 50 US multinational companies and 50 European multinational companies, of which 14 are state-owned enterprises, in 1970-1979 | European multinationals are less profitable than US ones because of the presence of state-owned enterprises among European multinationals. |
| Buckley, Clegg, Cross, Liu, Voss, and Zheng (2007) | JIBS | What are the determinants of Chinese outward foreign direct investment | Data on Chinese outward foreign direct investment, in 1984-2001 | Cultural proximity is an important determinant of internationalization for Chinese state-owned enterprises. The low cost of capital of Chinese state-owned enterprises allows them to invest in host countries with higher political risk. |
| Phelps (2007) | EG | What are the benefits from internationalization thorough state-owned enterprises | Case studies of Singapore–host country joint-venture industrial and technology parks in China, Vietnam, Indonesia, and India, involving government-linked companies | The economic benefits to Singapore from its overseas joint-venture parks have been modest. |
| Buckley, Cross, Tan, Xin, and Voss (2008) | MIR | Is Chinese outward foreign direct investment a Special Case of Emerging Country outward foreign direct investment? | Data on Chinese outward foreign direct investment | The strategic behavior of Chinese multinational companies (largely state-owned) is increasing, superseding behavior under classic internationalization theory. |
| García‐Canal and Guillén (2008) | SMJ | Risk and the strategy of foreign location choice of firms in regulated industries | 101 Latin American market entries of all listed Spanish firms in regulated industries, in 1987-2000 | Firms in regulated industries expand into countries characterized by governments with discretionary policymaking to be able to negotiate favorable conditions of entry. Minority-owned state enterprises exhibit a more tolerant attitude to risk. |
| Meyer and Altenborg (2008) | JIBS | How state-owned enterprises' national governance structures lead to incompatible strategies in a merge | A failed merger between two state-owned Scandinavian telecom firms | Cross-border acquisitions with good organizational fit can still fail because of strategic incompatibility. |
| Morck, Yeung, and Zhao (2008) | JIBS | What is the perspective on China's outward foreign direct investment | Not applicable | State-owned enterprises are the major players in China's outward foreign direct investment, and with their vast experience in navigating complex bureaucracies, they might do well in countries with similar institutional environments. |
| Rui and Yip (2008) | JWB | What is the strategic intent perspective of cross-border acquisitions led by Chinese firms | Lenovo, Nanjing Automobile, and Huawei | The internationalization of both state-owned enterprises and private firms is driven by a strategic intent perspective. |
| Cuervo-Cazurra and Dau (2009) | MIR | How do pro-market reforms affect firm exports | Latin American firms in 1990-2006 | Pro-market reforms support exports, but state-owned enterprises benefit less |
| Wan and Wong (2009) | JCF | Examines the stock price reaction of US oil companies to US sanction towards the acquisition by CNOOC (a Chinese state-owned enterprise) of Unocal (a US firm) | 66 US oil and gas exploration firms and 13 US oil refining firms | The shares of the US oil firms reacted unfavorably to the political sanction towards the acquisition by CNOOC (a Chinese state-owned enterprise) of Unocal (a US firm). |
| Chen and Young (2010) | APJM | How principal–principal conflicts in state-owned enterprises affect their international acquisition performance | 39 transactions undertaken by 32 Chinese publicly listed companies | Government ownership invokes a negative market reaction during a merger. Such negative effects are strengthened when state-owned enterprises operate in a competitive environment. |
| Kalotay and Sulstarova (2010) | JIM | What is the role of state ownership in Russian outward foreign direct investment | Cross-border acquisitions data with Russian firms as ultimate buyers | State-owned or -influenced multinational companies dominate Russian outward foreign direct investment to control the value chain. |
| Luo, Xue, and Han (2010) | MIR | What are the factors driving private firms from emerging markets to internationalize? | 1613 Chinese firms | Emerging market private firms that have acquired state-owned enterprises at home are more likely to internationalize. |
| Zhang, Zhou, and Ebbers (2011) | IBR | How do institutional factors influence the likelihood that Chinese overseas acquisition deals are completed? | 1324 announced Chinese cross-border acquisitions during 1982-2009 | Chinese state-owned acquirers lower the likelihood of completing cross-border acquisitions. |
| Cui and Jiang (2012) | JIBS | How state ownership affects Chinese firms’ foreign direct investment ownership | 132 foreign direct investment entries in 2002-2006 | State ownership increases home and host regulatory pressure on selecting joint ventures. |
| Duanmu (2012) | JWB | Impact of location characteristics on Chinese multinational company investments | 264 location choices by 189 Chinese multinational companies in 47 countries in 1999-2008 | State-owned enterprises are affected more by foreign exchange than private firms in location decisions. |
| Ramasamy, Yeung, and Laforet (2012) | JWB | What are the differences in international location decision between state-owned and private Chinese listed firms | 63 firms with foreign investments in 2006-2008 (1350 projects in 59 countries) | Central and local state-owned enterprises have more foreign direct investment projects in politically unstable countries than private firms. |
| Wang, Hong, Kafouros, and Wright (2012) | JIBS | How the government impacts the internationalization of firms from emerging markets | 626 Chinese firms with foreign direct investment from Annual Report of Industrial Enterprise Statistics and Chinese Ministry of Commerce | Higher-level government affiliation leads to more outward foreign direct investment and reinforces the impact of marketing and R&D investments. Government ownership does not lead to more outward foreign direct investment but reinforces the impact of R&D investments and efficiency. |
| Bass and Chakrabarty (2014) | JIBS | How multinationals acquire scarce resources | 404 cross-border transactions in the oil industry in 2005-2012 | State-owned enterprise investments are driven by natural resource security. |
| Choudhury and Khanna (2014) | JIBS | Why state-owned enterprises seek internationalization | 42 Indian state-owned laboratories from 1995 to 2006 | State-owned enterprises internationalize to gain resource independence from other state actors. |
| Colli, Mariotti, and Piscitello (2014) | JEPP | How does a country’s form of capitalism influences the internationalization of state-owned enterprises | Incumbents in the  energy and telecommunications industries in France, Germany, Italy, Spain, and the UK 1999-2013 | Coordinated market economies and state-influenced market economies promote internationalization, while liberal market economies act through *laissez-faire* policies. |
| Cuervo-Cazurra et al (2014) | JIBS | How the study of state-owned multinationals advances theory | Not applicable | State-owned multinationals ownership alters assumptions of models and can help extend theories by challenging assumptions. |
| Duanmu (2014) | JIBS | How host country expropriation risk affects investment decisions | 894 greenfield projects by Chinese firms | State-owned enterprises are less affected by expropriation risk in foreign investments. |
| Meyer, Ding, and Li (2014) | JIBS | How state-owned enterprises overcome discrimination abroad | 386 subsidiaries of Chinese listed state-owned and private firms in 2009 | State-owned enterprises use more acquisitions, especially in countries with less technology and less shareholder protection. |
| Zhang and He (2014) | IBR | How the evolution of regulations in light of the nationalism impact cross-border acquisitions | 7275 cross-border acquisition involved 76 countries during 1985-2010 | A good diplomatic relation between a foreign acquirer and the Chinese government increases the likelihood of completing cross-border acquisitions in China. |
| Du and Boateng (2015) | IBR | Whether state-owned enterprises create value for Chinese acquiring firms in cross-border acquisitions | 468 cross-border acquisitions during 1998-2011 | State ownership creates value in cross-border acquisitions, and liberalization of the foreign exchange approval system enhances state-owned enterprise’s internationalization by lowering the cost of doing business. |
| Lai, O'Hara, and Wysoczanska (2015) | APJM | What drives the internationalization of China’s two biggest national oil companies | Two Chinese national oil firms, Sinopec and CNPC, during 2002-2010 | Strategic assets and efficiency are the primary motives for Chinese state-owned outward foreign direct investments. |
| Liang, Ren, and Sun (2015) | JIBS | How state ownership and political ties affect internationalization before and after reform | 2394 publicly traded Chinese firms in 2001-2011 | Firms with some government control internationalize more after reform, and firms with political connections internationalize before the reform and less after the reform. |
| Wei, Clegg, and Ma (2015) | JIBS | How the Chinese government influences the internationalization of state-owned and private firms | Two state-owned enterprises and two private Chinese multinationals in 2008-2014 | Conscious government support of state-owned enterprises via finance and regulation enables state-owned enterprises internationalization and to become dominant players. Unconscious overprotection of state-owned enterprises helps private firms internationalize and to become market explorers. |
| Benito, Rygh, and Lunnan (2016) | GSJ | Comparison of benefits of internationalization | 30 publicly traded Norwegian firms in 2000-2010 | State-owned enterprises appear to benefit more from internationalization than private firms. |
| Estrin, Meyer, Nielsen, and Nielsen (2016) | JWB | Impact of institutions on state-owned firm internationalization | Matched sample of 153 majority-owned state-owned enterprises and 153 private publicly traded firms from 40 countries | Home country normative, regulatory, and governance controls induce state-owned enterprises to invest more abroad. |
| He, Eden, and Hitt (2016) | JIM | Institutional investors compensate for state ownership and lead to more internationalization | 253 state-owned multinationals in 42 countries in 2002-2007 | State-owned multinationals with institutional ownership have more international diversification in developed but not developing countries. |
| Liu, Gao, Lu, and Lioliou (2016) | JWB | Studies investment of firms from emerging markets in risky countries | 206 Chinese firms with investments in 58 foreign countries | State-owned enterprise localization is more sensitive to industry risk than private firms. |
| Rudy, Miller, and Wang (2016) | GSJ | What motivates state-owned enterprises to undertake foreign direct investment | Not applicable | Wholly owned state-owned enterprises invest abroad to obtain strategic assets and control supply. |
| Shi, Hoskisson, and Zhang (2016) | GSJ | What explains the opposition to foreign state-owned enterprises investments | Not applicable | Opposition to state-owned enterprises in the target country is weaker with geographic distance, religious similarity, political regime similarity, and resource complementarity, and these are weakened by nationalist politics. |
| Del Bo, Ferraris, and Florio (2017) | JCE | Does government ownership matter in the market for corporate control | 31,479 M&A deals in 138 countries in 2004-2012 | Deals involving state-owned enterprises are clearly different from the benchmark of private–private deals, due to the greater assets, higher solvency ratios, broader experience of deals, and closer proximity to targets of the acquirers. |
| Finchelstein (2017) | JWB | How different state actions support firm internationalization | 51 large firms from Argentina, Brazil, and Chile in 2015 | Direct actions and indirect actions support internationalization with different effects across countries because of the variety of public policies. |
| Hennart, Sheng, and Carrera Jr (2017) | JWB | How liberalization in Latin America led to state-owned multinationals | 173 firms in 2002-2011 | Government ownership leads to higher internationalization through direct ownership and indirect ownership via investment bank and pension funds, reinforced by family ownership. |
| Huang, Xie, Li, and Reddy (2017) | IBR | How state ownership supports outward foreign direct investment | 507 publicity traded Chinese manufacturing firms in 2007-2013 | State-owned enterprises have fewer new foreign subsidiaries, especially central state-owned enterprises, but more if facing more institutional development and market competition. |
| Karolyi and Liao (2017) | JCF | What are the drivers and consequences of cross-border acquisitions led by government-controlled acquirers | 27786 announced cross-border acquisitions during 1990 - 2008 with a total deal value equal to USD9.04 trillion | The motives behind the cross-border acquisitions led by government-controlled acquirers differ little from those by corporate acquirers. Short-term market reaction towards deals led by government-controlled acquirers are similar to those by corporate acquirers. |
| Li, Xia, and Lin (2017) | SMJ | How state ownership affects the completion of cross-border M&As | Matched sample of 914 cross-border acquisitions in the US in 1990-2012 | State-owned enterprises complete more cross-border deals than private firms, but less if target is public, in R&D alliance and more if state-owned enterprises have acquisition and alliance experiences in host countries; but no difference in duration of completion. |
| Pinto, Ferreira, Falaster, Fleury, and Fleury (2017) | JWB | How does government support affect ownership in cross-border acquisitions | 262 cross-border acquisitions by Brazilian firms in 2006-2012 | Government stock and political ties individually lead to full acquisitions. |
| Xie and Li (2017) | APJM | How emerging market multinationals imitate each other entry mode | 608 cross-border acquisitions by Chinese firms from 1987 to 2008 | Previous home country majority acquisitions lead to more majority acquisitions; previous private majority Chinese acquisitions lead to more state-owned enterprises’ majority acquisitions, and previous state-owned enterprises’ majority acquisitions lead to less state-owned enterprises’ majority acquisitions. |
| Arreola and Bandeira-de-Mello (2018) | MIR | How minority ownership types affect internationalization | 38 Brazilian traded multinationals in 2006-2010 and one case study of sugar/ethanol firm | State institutional investors lead to more internationalization and state ownership leads to less internationalization. |
| Cannizzaro and Weiner (2018) | JIBS | Drivers of transparency in outward foreign direct investment | 965 investment in the oil industry in 2000-2011 | State ownership reduces transparency but less if the home country is better governed and state-owned enterprises are less sensitive to political risk. |
| Chen, Musacchio, and Li (2019) | JM | How do principal-principal conflicts amongst blockholders impact the cross-border acquisition activity of state-owned enterprises | 7,564 cross-border acquisitions in 2004-2013 | Conflicts among different blockholders in state-owned enterprises make it difficult to pursue large-scale, cross-border deals. |
| Buckley, Clegg, Voss, Cross, Liu, and Zheng (2018) | JIBS | A review and agenda for future research on international investments by Chinese state-owned enterprises and multinationals | Not applicable | Earlier internationalization was allowed only for state-owned enterprises (1984-2001), driven by market and resource considerations. In later periods, the Chinese government continued to influence the selection of host countries for investment by multinationals, both state-owned and private. This was facilitated by China’s foreign trade agreements with host countries. The internationalization of state-owned enterprises aligned with China’s imperatives, but limited direct benefits and spillovers in the host country |
| Clegg, Voss, and Tardios (2018) | JWB | How the home country autocracy affects state-owned enterprises internationalization | 1386 state-owned enterprises' and 136146 private cross-border acquisitions in 1996-2015 in 25 countries | State-owned enterprises from autocratic home countries make more cross-border acquisitions, especially in other autocratic countries. |
| De Beule, Somers, and Zhang (2018) | MIR | Where do Chinese manufacturing firms locate greenfield in Europe | 565 greenfield investments by 374 Chinese firms in 2004-2012 in European Union | Previous investments by private firms in the same and other sectors drive new investments in the region. |
| Deng, Yan, and van Essen (2018) | IBR | Impact of political connections on outward foreign direct investment | Datasets with outward foreign direct investment from the Ministry, merged with data on publicly traded firms | State-owned enterprises less likely to invest abroad and private firms with political connections more likely to invest abroad. |
| Li, Cui, and Lu (2018) | JIBS | How central and local state-owned enterprises differ in their internationalization | Not applicable | Central state-owned enterprises are subject to more legitimacy pressures that affect their internationalization path, diversification, and entry method. |
| Li, Xia, Shapiro, and Lin (2018) | JWB | How state ownership affects foreign direct investment | 2585 Chinese firms with 1454 foreign subsidiaries in 2001-2014 | State-owned enterprises have fewer foreign subsidiaries, but more if the province has more market forces and other state-owned enterprises have foreign direct investment. |
| Rodrigues and Dieleman (2018) | JWB | How state ownership affects internationalization | Brazilian mining firm Vale 2008-2015 interviews | State-owned enterprises internationalize to reduce dependence but the creation of additional dependence and codependence with the government leads to a reduction in internationalization. |
| Zhou (2018) | MIR | How do hybrid state-owned enterprises internationalize | 1600 listed Chinese state-owned (more than 5%) firms in 1991-2016 | Majority state-owned enterprises have more foreign direct investment, reinforce the impact of ‘go global’ campaign and debt; and weaken the impact of intangible assets on number of foreign direct investments. |
| Grøgaard, Rygh, and Benito (2019) | JIBS | Whether entry mode  decisions of state-owned enterprises differ from privately owned enterprises | Transactions of assets and firms in the Canadian oil and gas industry over 2005-2016 | Compared to private firms, state-owned enterprises tend to prefer acquiring stand-alone assets rather than firms, and to take lower ownership shares. The differences between state-owned enterprises and private firms diminish when home countries are characterized by high government quality and market orientation. |
| Li, Li, and Wang (2019) | SMJ | How state ownership affects cross-border acquisitions completion outcomes | 1,170 deals from 1990 and 2010 | The deal completion rate is 14% lower for state-owned enterprises than non-state-owned enterprises, and opaqueness aggravates the negative relationship between state-owned acquirers and deal completion. |
| Mariotti and Marzano (2019) | JIBS | Whether entry mode decisions of state-owned enterprises differ from privately owned enterprises | Panel dataset of 99 firms over 1995-2014 | State-controlled enterprises internationalize more (less) than privately owned enterprises in coordinated (liberal) market economies, whereas they exhibit inconsistent behavior in state-influenced market economies. |
| Parente, Rong, Geleilate, and Misati (2019) | JIBS | How multinational companies sustain operations in challenging countries | Weihai Chinese state-owned enterprise in Democratic Republic of Congo 2011 to 2018 | State-owned enterprises succeed in challenging countries when the home government supports negotiation and resource access, with firms internationalizing by relying on the ethnic network before building a local network. |
| Aguilera, Duran, Heugens, Sauerwald, Turturea, and VanEssen (2020) | JWB | When does state ownership improve firm financial performance | Meta-analysis of studies spanning 53 years and 131 countries | The political ideology of the government, both independently and in conjunction with political institutions (state capacity and political constraint), affects the impact of state ownership on the performance of state-owned enterprises. |
| Cheung, Aalto, and Nevalainen (2020) | JWB | How does a shift in dominant institutional logic from state to market logic affect SOEs’ evaluation of international venture opportunities | In-depth historical case study of state-owned Telecom Finland, based on 54,000 pages of primary sources over 1987-1998 | Although state-owned firms can internationalize to the same extent or even more than private firms, different rationales underline their internationalization, and these rationales might change during long internationalization processes, influencing geographical and partner preferences. |
| Lazzarini, Mesquita, Monteiro, and Musacchio (2020) | JIBS | Are state-owned enterprises better at invention than private firms | Patent inventions of 521 state-owned enterprises and matched private firms across 43 countries, over 1997–2012 | Patenting by state-owned enterprises at a base level is more pioneering and frequent than that of private firms. |
| Sun, Deng, and Wright (2020) | JIBS | Examining how host state ownership affects both  innovation inputs and outputs in China-based international joint ventures | International joint-ventures in the Chinese manufacturing sector over 2008–2013 | R&D investment can be politically motivated and symbolically managed to  Ensure continued resource exchanges with the host state. |
| Wang, Kafouros, Yi, Hong, and Ganotakis (2020) | JWB | What is the role of government affiliation in firm innovativeness and profitability in emerging countries | 18,430 Chinese firms over 2005-2007 | Affiliation with higher-level governments enhances firms’ innovativeness, whereas affiliation with lower-level governments is effective for enhancing profitability. |
| Cuervo-Cazurra and Li (2021) | JWB | Disentangling theoretical predictions of the advantage and disadvantage of stateness relating to the internationalization of state-owned enterprises | Systematic content analysis of 83 articles on state-owned multinationals | Governments seem to induce state-owned multinationals to select more challenging countries. Alignment between firm strategies and national interests is a deciding effect in the location choices of state-owned multinationals. Cross-border acquisitions seem to result in adverse reactions. |
| Wright, Wood, Musacchio, Okhmatovskiy, Grosman, and Doh (2021) | JWB | What factors represent key dimensions of state capitalism | Exploratory factor analysis of 59 countries and seven variables characterizing direct state involvement in economic activities (2014) | Framework of eight initial varieties of state capitalism according to three dimensions of state capitalism —the level of government threat, state ownership, and statism. |

Journals: Asia Pacific Journal of Management (APJM), Global Strategy Journal (GSJ), International Business Review (IBR), Journal of Economic Geography (JEG), Journal of Comparative Economics (JCE), Journal of European Public Policy (JEPP), Journal of International Business Studies (JIBS), Journal of International Management (JIM), Journal of Management (JM), Journal of World Business (JWB), Management and Organizational Review (MOR), and Management International Review (MIR), Academy of Management Journal (AMJ), Academy of Management Review (AMR), Administrative Science Quarterly (ASQ), American Economic Review (AER), Journal of Finance (JF), Journal of Financial Economics (JFE), Journal of Political Economy (JPE), Management Science (MS), Organization Science (OS), Quarterly Journal of Economics (QJE), and Strategic Management Journal (SMJ). Adadpted and extended from Cuervo-Cazurra & Li (2021)

Table B. Summary of selected studies on the internationalization of sovereign wealth funds

| **Authors (Year)** | **Journal** | **Research Question** | **Sample** | **Main Arguments and Findings** |
| --- | --- | --- | --- | --- |
| Hebb and Wójcik (2005) | EAPA | What is the investment strategy of global pension funds in emerging markets | Case study of California Public Employees  Retirement System (CalPERS) | In order to mitigate the risks posed by poor  corporate standards of behavior, institutional investors increasingly apply nonfinancial criteria not only to individual firms in emerging markets, but to the corporate practices of whole countries, by demanding levels of corporate and social behavior greater than those currently  consistent with countries' regulatory frameworks. |
| Clark (2008) | EG | What are the criteria for well-governed pension funds internationally | Two public pension funds and two corporate pension funds, all anonymized | There is a premium on good governance in institutions that rely upon financial markets for long-term objectives; and a global market for governance principles and practices. |
| Dixon (2008) | NPE | The evolution of French state pension system | Not applicable | The French pension system is converging towards the Anglo-American funded model as a response to increased globalization. |
| Eaton and Ming (2010) | RIPE | How the Chinese government influences the sovereign wealth fund investment and competitive strategies | China  Investment Corporation (CIC), world’s third largest sovereign wealth fund | The sovereign wealth fund was drawn into competition for high yield, high-risk investments with the government foreign exchange reserve management agency. CIC’s investments were mostly in the domestic bank sector. |
| Gospel, Pendleton, Vitols, and Wilke (2011) | CGIR | What are the consequences for employment when companies are acquired by private equity, hedge funds, or sovereign wealth funds | Three case studies–a Spanish supermarket chain, a German engineering company, and a UK ports and logistics group | Greater disclosure and regulation of sovereign wealth funds are more likely to enhance employee protection than further labor regulation. |
| Haberly (2011) | EAPA | How do governments use sovereign wealth funds as tools to promote national development | Two case studies: Qatar, Abu Dhabi, and Dubai's use of sovereign wealth funds to promote the development of their aerospace sectors; and the deployment of the China Investment Corporation as an instrument of Chinese raw materials and energy policy. | Strategically oriented sovereign wealth fund investment is used by the governments to adapt to globalization and financialization. The viability of such a strategy hinges on the way it feeds into the strategies of target firms and host governments. |
| Kotter and Lel (2011) | JFE | What are the target selection decisions of sovereign wealth funds | Sovereign wealth funds’ 417 investments in 326 firms (some of them receiving multiple investments) in 1980-2009 | Sovereign wealth funds prefer multinational  firms, likely due to existing business relations with them or diversification benefits of multinationals. Sovereign wealth funds appear to invest in developed countries. |
| Monk (2011) | EAPA | Why sovereign wealth funds exist | Not applicable | Sovereign wealth funds exist to preserve local autonomy and state sovereignty by harnessing the power of finance as a response to globalization and financialization. The purposes of sovereign wealth funds include self-insurance, macroeconomic stability, budget planning and stabilization, and long-term commitment to future welfare. |
| Knill, Lee, and Mauck (2012) | JCF | What is the role of bilateral political relations in sovereign wealth funds investment decisions | Over 900 acquisitions of public and private targets by sovereign wealth funds over the period 1984–2009 | Contrary to predictions based on the foreign direct investment and political relations literature, political relations matter less for sovereign wealth funds in determining how much to invest. Sovereign wealth fund investment has a positive impact on relatively closed countries. |
| Knill, Lee, and Mauck (2012) | JFI | What is the relationship between sovereign wealth  fund investment and the return-to-risk performance of target firms | 130 acquisitions by sovereign wealth funds from the inception of each fund and up to 2009 | In cases of foreign investment, sovereign wealth funds’ target firm performance is more like other state-owned firms. |
| Bernstein, Lerner, and Schoar (2013) | JEP | How institutional arrangements influence the investment decision policies of sovereign wealth funds, what is the impact of politicians involved in fund management | 29 sovereign wealth funds that carried out 2,662 transactions in 1984-2007 | Sovereign wealth funds with politician involvement are more likely to invest domestically, while those sovereign wealth funds where external managers play an important role are more likely to invest internationally. Funds with more politically connected board members underperform. |
| Johan, Knill, and Mauck (2013) | JIBS | What is the extent to which sovereign wealth funds resemble other institutional investors when it comes to the choice between private and public cross-border investment | The investments of 19 sovereign wealth funds in 424 public and private firms around the world, over 1991- 2010 | Unlike other institutional investors, sovereign wealth funds are more likely to invest in private equity compared with public equity in countries where investor protection is low, and where the bilateral political relations are weak. |
| Megginson, You, and Han (2013) | FR | Country-level determinants of sovereign wealth fund cross-border investments | 1,590 acquisitions by sovereign wealth funds from 15 countries in listed firms in 78 target countries over 1985-2011 | Sovereign wealth funds from countries with high levels of openness and economic development, but with less developed capital markets, will make more cross-country transactions, while target countries with higher levels of investor protection and more developed capital markets will attract more sovereign wealth fund investment. |
| Vasudeva (2013) | OS | Why responsible investment constitutes a central feature of Norway’s Government Pension Fund Global investment strategy. To what extent Norway’s Fund serves as an instrument of the Norwegian state in shaping behavioral norms for Norwegian firms | Data on cross-border investments for 437 Norwegian firms in 49 industries over 1999–2010. | By making responsible investments core of its cross-border investment strategy, Norway’s Government Pension Fund Global seeks to establish legitimacy both at home and internationally. The normative pressure for responsible investments is mediated by firms’ imitation of the Fund’s investments, but this effect becomes weaker for state-owned enterprises. |
| Bertoni and Lugo (2014) | JCF | What is the effect of sovereign wealth funds on the credit risk of their  portfolio companies | Credit default swap spreads of 391 investments by sovereign wealth funds in 198 firms in 1984-2010 | Political factors significantly affect the magnitude of the decrease in credit risk: sovereign wealth funds from authoritarian countries reduce the credit risk of their investments, and this effect is offset in some cases by the opacity of sovereign wealth funds. The political stability of a home country and a moderate bilateral political relationship also reduce the credit risk. |
| Haberly (2014) | EG | The interaction of home and host countries’ institutions through foreign investments by sovereign wealth funds | Analysis of Gulf Cooperation Council sovereign wealth fund  investment in German industry after the financial crisis of 2008 | German industrial firms, particularly the major automotive firms, seek long-term sovereign wealth funds investment as an adaptive response to the stresses of financial restructuring, and as a pre-emptive measure against hostile takeovers. |
| Bortolotti, Fotak, and Megginson (2015) | RFS | How do sovereign wealth funds perform | 33 sovereign wealth funds from 21 countries, domestic and international investments made by sovereign wealth funds, and by sovereign wealth fund majority-owned subsidiaries over 1980-2012. | Cumulative abnormal returns of sovereign wealth fund equity investments in publicly traded firms are positive but lower than those of comparable private investments. Sovereign wealth fund targets suffer from declining return on assets and sales growth over the following three years. Larger discounts are associated with sovereign wealth funds taking seats on boards and with sovereign wealth funds under strict government control acquiring greater stakes. |
| Aguilera, Capapé, and Santiso (2016) | AMP | Strategic governance topology of sovereign wealth funds | Not applicable | As a response to agency and legitimacy-related conflicts, strategic governance of sovereign wealth funds is organized into 1) shareholder activism, 2) setting up in-house capabilities, 3) acquiring legitimacy as institutional investors, and 4) long-term learning to engage in country-to-country relations. |
| Boubakri, Cosset, and Grira (2016) | JIFMIM | Compare sovereign wealth funds investment decisions and target selection with pension funds | 344 firms targeted by sovereign wealth funds in 1991-2011 to compare to 663 firms targeted by pension funds | Sovereign wealth funds are more likely to target profitable firms in strategic sectors and in countries with higher economic growth and weaker legal and institutional environment. |
| Calluzzo, Dong, and Godsell (2017) | JIBS | Investigate if sovereign wealth funds are attracted by the US  campaign finance firms to gain access to the US  political process | 2036 unique sovereign wealth fund investments made in 1755 publicly listed US firms over 2000-2014 | Sovereign wealth funds are increasingly attracted by the US campaign finance firms after the legislative shock liberalizing corporate campaign finance activity. These campaign firms increase their political contributions after receiving sovereign wealth fund investments. |
| Debarsy, Gnabo, and Kerkour (2017) | JIMF | Identify the driving forces of cross-border investments emanating from sovereign wealth funds and test the existence of spatial competition among host countries. | Annual cross-border sovereign wealth fund net inflows for 43 host countries over the period 2004–2009 | Countries with higher gross domestic product per capita and domestic flows attract more sovereign wealth funds capital. Better political stability and higher level of financial development of host country contribute positively to sovereign wealth fund’s investments net flows, while stock market volatility has the opposite effect. |
| Aggarwal and Goodell (2018) | IBR | What are the determinants of sovereign wealth fund governance | Data on 49 large sovereign wealth funds from 33 countries | National culture matters greatly in determining sovereign wealth fund governance. Sovereign wealth fund governance is negatively associated with the national cultural dimension of power distance and individualism; and positively associated with the national cultural dimensions of long-term orientation and uncertainty avoidance. |
| Vasudeva, Nachum, and Say (2018) | AMJ | Signaling effect of sovereign wealth fund investments on the level of equity ownership in foreign acquisition targets | Norway’s Government Pension Fund Global foreign investments in 47 countries by firms from Norway (559 firms) and Sweden (1,256 firms) over 1998–2011 | Activist sovereign wealth fund can serve as an intermediary, signaling about the quality of host countries’ institutional environment to internationalizing firms. |
| Goergen, O'Sullivan, Wood, and Baric (2018) | HRMJ | What is the impact of equity ownership in UK listed firms by the Norwegian Government Pension Fund‐Global on labor demand in these firms | 508 firms with Norway’s Government Pension Fund Global ownership for at least one year over 2006–2013 | Firms in which Norway’s Government Pension Fund Global has invested are significantly less likely to reduce their demand for labor, more specifically in the immediate aftermath of the 2008 financial crisis. When a drop in the demand for labor occurs, it is less extreme compared to similar organizations without Norway’s Government Pension Fund Global shareholding. |
| Liu, Mauck, and Price (2019) | JREFE | Examining sovereign wealth fund investment in real  estate | 856 real estate purchases by sovereign wealth funds and public pension funds over 1974-2016 | Sovereign wealth funds are significantly more likely than public pension funds to invest across international borders. The percentage of sovereign wealth funds’ cross-border real estate investment is substantially higher than the percentage of sovereign wealth funds cross-border investment in public and private equity. |
| Park, Xu, In, and Ji (2019) | JFM | Examining the long-term impact of sovereign wealth fund investments on firm value | 709 sovereign wealth fund transactions over 1989-2015 | Sovereign wealth fund investments have a negative effect on the value of target firms. This effect depends on the level of investor protection in host countries. Domestic sovereign wealth funds impart a stabilizing effect on the stock price of target firms in countries with low investor protection, but the effect is weaker for foreign sovereign wealth funds. |
| Bahoo, Alon, and Paltrinieri (2020) | IRFA | What are the key research streams in the sovereign wealth funds literature? | Meta-literature review, covering 184 articles over 2005-2019 | Articles about investment strategies of sovereign wealth funds constitute the bulk of the research. The vast majority of studies maintain that their investments are associated with political connections. |

Journals: Academy of Management Journal (AMJ), Academy of Management Perspectives (AMP), Corporate Governance: An International Review (CGIR), Environment and Planning A (EAPA), Economic Geography (EG), Financial Review (FR), Human Resource Management Journal (HRMJ), International Business Review (IBR), Journal of Corporate Finance (JCF), Journal of Economic Perspectives (JEP), Journal of Financial Economics (JFE), Journal of Financial Intermediation (JFI), Journal of Financial Markets (JFM), Journal of International Business Studies (JIBS), Journal of International Financial Markets, Institutions & Money (JIFMIM), Journal of International Money and Finance (JIMF), Journal of Real Estate Finance and Economics (JREFE), Organization Science (OS), Review of Financial Studies (RFS), Review of International Political Economy (RIPE).

**REFERENCES**

Aggarwal, R. & Goodell, J. W. 2018. Sovereign wealth fund governance and national culture. *International Business Review*, 27(1): 78-92.

Aguilera, R., Duran, P., Heugens, P. P. M. A. R., Sauerwald, S., Turturea, R., & VanEssen, M. 2020. State ownership, political ideology, and firm performance around the world. *Journal of World Business*.

Aguilera, R. V., Capapé, J., & Santiso, J. 2016. Sovereign wealth funds: A strategic governance view. *The Academy of Management Perspectives*, 30(1): 5-23.

Aharoni, Y. 1980. The state owned enterprise as a competitor in international markets. *Columbia Journal of World Business*, 15(1): 14-22.

Arreola, F. & Bandeira-de-Mello, R. 2018. The differential effects of minority state ownership types on the internationalization of emerging market multinationals from democratic states. *Management International Review*, 58(5): 845-69.

Bahoo, S., Alon, I., & Paltrinieri, A. 2020. Sovereign wealth funds: Past, present and future. *International Review of Financial Analysis*, 67: 101418.

Bass, A. E. & Chakrabarty, S. 2014. Resource security: Competition for global resources, strategic intent, and governments as owners. *Journal of International Business Studies*, 45(8): 961-79.

Benito, G. R., Rygh, A., & Lunnan, R. 2016. The benefits of internationalization for state‐owned enterprises. *Global Strategy Journal*, 6(4): 269-88.

Bernstein, S., Lerner, J., & Schoar, A. 2013. The investment strategies of sovereign wealth funds. *Journal of Economic Perspectives*, 27(2): 219-38.

Bertoni, F. & Lugo, S. 2014. The effect of sovereign wealth funds on the credit risk of their portfolio companies. *Journal of Corporate Finance*, 27: 21-35.

Bortolotti, B., Fotak, V., & Megginson, W. L. 2015. The sovereign wealth fund discount: Evidence from public equity investments. *The Review of Financial Studies*, 28(11): 2993-3035.

Boubakri, N., Cosset, J.-C., & Grira, J. 2016. Sovereign wealth funds targets selection: A comparison with pension funds. *Journal of International Financial Markets, Institutions and Money*, 42: 60-76.

Buckley, P. J., Clegg, L. J., Cross, A., Liu, X., Voss, H., & Zheng, P. 2007. The determinants of Chinese outward foreign direct investment. *Journal of International Business Studies*, 38(4): 499-518

Buckley, P. J., Clegg, L. J., Voss, H., Cross, A. R., Liu, X., & Zheng, P. 2018. A retrospective and agenda for future research on Chinese outward foreign direct investment. *Journal of International Business Studies*, 49(1): 4-23.

Buckley, P. J., Cross, A. R., Tan, H., Xin, L., & Voss, H. 2008. Historic and emergent trends in Chinese outward direct investment. *Management International Review*, 48(6): 715-48.

CABS. 2019. Academic Journal Guide 2018: Chartered Association of Business Schools.

Calluzzo, P., Dong, G. N., & Godsell, D. 2017. Sovereign wealth fund investments and the US political process. *Journal of International Business Studies*, 48(2): 222-43.

Canabal, A. & White III, G. O. 2008. Entry mode research: Past and future. *International Business Review*, 17(3): 267-84.

Cannizzaro, A. P. & Weiner, R. J. 2018. State ownership and transparency in foreign direct investment. *Journal of International Business Studies*, 49(2): 172-95.

Chen, V. Z., Musacchio, A., & Li, S. 2019. A principals-principals perspective of hybrid leviathans: cross-border acquisitions by state-owned MNEs. *Journal of Management*, 45(7): 2751-78

Chen, Y. Y. & Young, M. N. 2010. Cross-border mergers and acquisitions by Chinese listed companies: A principal–principal perspective. *Asia Pacific Journal of Management*, 27(3): 523-39.

Cheung, Z., Aalto, E., & Nevalainen, P. 2020. Institutional Logics and the Internationalization of a State-Owned Enterprise: Evaluation of International Venture Opportunities by Telecom Finland 1987–1998. *Journal of World Business*, 55(6).

Choudhury, P. & Khanna, T. 2014. Toward resource independence–Why state-owned entities become multinationals: An empirical study of India’s public R&D laboratories. *Journal of International Business Studies*, 45(8): 943-60.

Clark, G. L. 2008. Governing finance: Global imperatives and the challenge of reconciling community representation with expertise. *Economic Geography*, 84(3): 281-302.

Clegg, L. J., Voss, H., & Tardios, J. A. 2018. The autocratic advantage: Internationalization of state-owned multinationals. *Journal of World Business*, 53(5): 668-81.

Colli, A., Mariotti, S., & Piscitello, L. 2014. Governments as strategists in designing global players: the case of European utilities. *Journal of European Public Policy*, 21(4): 487-508.

Cuervo-Cazurra, A. & Dau, L. A. 2009. Structural reform and firm exports. *Management International Review*, 49(4): 479-507.

Cuervo-Cazurra, A. & Li, C. 2021. State ownership and internationalization: The advantage and disadvantage of stateness. *Journal of World Business*, 56: 101112.

Cui, L. & Jiang, F. 2012. State ownership effect on firms' FDI ownership decisions under institutional pressure: a study of Chinese outward-investing firms. *Journal of International Business Studies*, 43(3): 264-84.

De Beule, F., Somers, D., & Zhang, H. 2018. Who follows whom? A location study of Chinese private and state-owned companies in the European Union. *Management International Review*, 58(1): 43-84.

Debarsy, N., Gnabo, J.-Y., & Kerkour, M. 2017. Sovereign wealth funds’ cross-border investments: Assessing the role of country-level drivers and spatial competition. *Journal of International Money and Finance*, 76: 68-87.

Del Bo, C. D., Ferraris, M., & Florio, M. 2017. Governments in the market for corporate control: Evidence from M&A deals involving state-owned enterprises. *Journal of Comparative Economics*, 45(1): 89-109.

Deng, Z., Yan, J., & van Essen, M. 2018. Heterogeneity of political connections and outward foreign direct investment. *International Business Review*, 27(4): 893-903.

Dixon, A. D. 2008. The rise of pension fund capitalism in Europe: an unseen revolution? *New Political Economy*, 13(3): 249-70.

Du, M. & Boateng, A. 2015. State ownership, institutional effects and value creation in cross-border mergers & acquisitions by Chinese firms. *International Business Review*, 24(3): 430-42.

Duanmu, J.-L. 2012. Firm heterogeneity and location choice of Chinese multinational enterprises (MNEs). *Journal of World Business*, 47(1): 64-72.

Duanmu, J.-L. 2014. State-owned MNCs and host country expropriation risk: The role of home state soft power and economic gunboat diplomacy. *Journal of International Business Studies*, 45(8): 1044-60.

Duriau, V. J., Reger, R. K., & Pfarrer, M. D. 2007. A content analysis of the content analysis literature in organization studies: Research themes, data sources, and methodological refinements. *Organizational Research Methods*, 10(1): 5-34.

Eaton, S. & Ming, Z. 2010. A principal–agent analysis of China's sovereign wealth system: Byzantine by design. *Review of International Political Economy*, 17(3): 481-506.

Estrin, S., Meyer, K. E., Nielsen, B. B., & Nielsen, S. 2016. Home country institutions and the internationalization of state owned enterprises: A cross-country analysis. *Journal of World Business*, 51(2): 294-307.

Finchelstein, D. 2017. The role of the State in the internationalization of Latin American firms. *Journal of World Business*, 52(4): 578-90.

Franko, L. G. 1975. Patterns in the multinational spread of Continental European enterprise. *Journal of International Business Studies*: 41-53.

García‐Canal, E. & Guillén, M. F. 2008. Risk and the strategy of foreign location choice in regulated industries. *Strategic Management Journal*, 29(10): 1097-115.

Gaur, A. & Kumar, M. 2018. A systematic approach to conducting review studies: An assessment of content analysis in 25 years of IB research. *Journal of World Business*, 53(2): 280-89.

Geyskens, I., Krishnan, R., Steenkamp, J.-B. E., & Cunha, P. V. 2009. A review and evaluation of meta-analysis practices in management research. *Journal of Management*, 35(2): 393-419.

Goergen, M., O'Sullivan, N., Wood, G., & Baric, M. 2018. Sovereign wealth funds, productivity and people: The impact of Norwegian Government Pension Fund‐Global investments in the United Kingdom. *Human Resource Management Journal*, 28(2): 288-303.

Gospel, H., Pendleton, A., Vitols, S., & Wilke, P. 2011. New investment funds, restructuring, and labor outcomes: A European perspective. *Corporate Governance: An International Review*, 19(3): 276-89.

Grøgaard, B., Rygh, A., & Benito, G. R. 2019. Bringing corporate governance into internalization theory: State ownership and foreign entry strategies. *Journal of International Business Studies*.

Haberly, D. 2014. White knights from the gulf: Sovereign wealth fund investment and the evolution of German industrial finance. *Economic Geography*, 90(3): 293-320.

He, X., Eden, L., & Hitt, M. A. 2016. Shared governance: Institutional investors as a counterbalance to the state in state owned multinationals. *Journal of International Management*, 22(2): 115-30.

Hebb, T. & Wójcik, D. 2005. Global standards and emerging markets: the institutional-investment value chain and the CalPERS investment strategy. *Environment and Planning A*, 37(11): 1955-74.

Hennart, J.-F., Sheng, H. H., & Carrera Jr, J. M. 2017. Openness, international champions, and the internationalization of Multilatinas. *Journal of World Business*, 52(4): 518-32.

Huang, Y., Xie, E., Li, Y., & Reddy, K. 2017. Does state ownership facilitate outward FDI of Chinese SOEs? Institutional development, market competition, and the logic of interdependence between governments and SOEs. *International Business Review*, 26(1): 176-88.

Johan, S. A., Knill, A., & Mauck, N. 2013. Determinants of sovereign wealth fund investment in private equity vs public equity. *Journal of International Business Studies*, 44(2): 155-72.

Kalotay, K. & Sulstarova, A. 2010. Modelling Russian outward FDI. *Journal of International Management*, 16(2): 131-42.

Knill, A., Lee, B.-S., & Mauck, N. 2012. Bilateral political relations and sovereign wealth fund investment. *Journal of Corporate Finance*, 18(1): 108-23.

Knill, A. M., Lee, B. S., & Mauck, N. 2012. Sovereign wealth fund investment and the return-to-risk performance of target firms. *Journal of Financial Intermediation*, 21(2): 315-40.

Kotter, J. & Lel, U. 2011. Friends or foes? Target selection decisions of sovereign wealth funds and their consequences. *Journal of Financial Economics*, 101(2): 360-81.

Lai, H., O'Hara, S., & Wysoczanska, K. 2015. Rationale of internationalization of China's national oil companies: seeking natural resources, strategic assets or sectoral specialization? *Asia Pacific Business Review*, 21(1): 77-95.

Lazzarini, S. G., Mesquita, L. F., Monteiro, F., & Musacchio, A. 2020. Leviathan as an inventor: An extended agency model of state-owned versus private firm invention in emerging and developed economies. *Journal of International Business Studies*: 1-35.

Li, J., Li, P., & Wang, B. 2019. The liability of opaqueness: State ownership and the likelihood of deal completion in international acquisitions by Chinese firms. *Strategic Management Journal*, 40(2): 303-27.

Li, J., Xia, J., & Lin, Z. 2017. Cross‐border acquisitions by state‐owned firms: How do legitimacy concerns affect the completion and duration of their acquisitions? *Strategic Management Journal*, 38(9): 1915-34.

Li, J., Xia, J., Shapiro, D., & Lin, Z. 2018. Institutional compatibility and the internationalization of Chinese SOEs: The moderating role of home subnational institutions. *Journal of World Business*, 53(5): 641-52.

Li, M. H., Cui, L., & Lu, J. 2018. Varieties in state capitalism: Outward FDI strategies of central and local state-owned enterprises from emerging economy countries, *State-Owned Multinationals*: Journal of International Business Studies Special Collections.

Liang, H., Ren, B., & Sun, S. L. 2015. An anatomy of state control in the globalization of state-owned enterprises. *Journal of International Business Studies*, 46(2): 223-40.

Lipsey, M. W. & Wilson, D. B. 2001. *Practical meta-analysis*: Sage Publications, Inc.

Liu, P., Mauck, N., & Price, M. 2019. Are government owned investment funds created equal? Evidence from sovereign wealth fund real estate acquisitions. *Journal of Real Estate Finance and Economics*.

Liu, X., Gao, L., Lu, J., & Lioliou, E. 2016. Environmental risks, localization and the overseas subsidiary performance of MNEs from an emerging economy. *Journal of World Business*, 51(3): 356-68.

Luo, Y., Xue, Q., & Han, B. 2010. How emerging market governments promote outward FDI: Experience from China. *Journal of World Business*, 45(1): 68-79.

Mariotti, S. & Marzano, R. 2019. Varieties of capitalism and the internationalization of state-owned enterprises. *Journal of International Business Studies*, 50(5): 669-91.

Mazzolini, R. 1980. European Government-Controlled Enterprises: An Organizational Political View. *Journal of International Business Studies*, 11(1): 48-58.

Mazzolini, R. 1979. *Government controlled enterprises: International strategic and policy decisions*. Hoboken, NJ: John Wiley & Sons.

Mazzolini, R. 1980. The international strategy of state‐owned firms: An organizational process and politics perspective. *Strategic Management Journal*, 1(2): 101-18.

Megginson, W. L., You, M., & Han, L. 2013. Determinants of sovereign wealth fund cross‐border investments. *Financial Review*, 48(4): 539-72.

Meyer, C. B. & Altenborg, E. 2008. Incompatible strategies in international mergers: The failed merger between Telia and Telenor. *Journal of International Business Studies*, 39(3): 508-25.

Meyer, K. E., Ding, Y., & Li, J. 2014. Overcoming distrust: How state-owned enterprises adapt their foreign entries to institutional pressures abroad. *Journal of International Business Studies*, 45(8): 1005-28.

Monk, A. H. 2011. Sovereignty in the era of global capitalism: the rise of sovereign wealth funds and the power of finance. *Environment and Planning A*, 43(8): 1813-32.

Morck, R., Yeung, B., & Zhao, M. 2008. Perspectives on China's outward foreign direct investment. *Journal of International Business Studies*, 39(3): 337-50.

Ormans, L. 2016. 50 Journals used in FT Research Rank. Financial Times, September 12. Accessed November 11, 2019: Financial Times.

Parente, R., Rong, K., Geleilate, J.-M. G., & Misati, E. 2019. Adapting and sustaining operations in weak institutional environments: A business ecosystem assessment of a Chinese MNE in Central Africa. *Journal of International Business Studies*, 50(2): 275-91.

Park, R. J., Xu, S., In, F., & Ji, P. I. 2019. The long-term impact of sovereign wealth fund investments. *Journal of Financial Markets*, 45: 115-38.

Phelps, N. A. 2007. Gaining from globalization? State extraterritoriality and domestic economic impacts—the case of Singapore. *Economic Geography*, 83(4): 371-93.

Pinto, C. F., Ferreira, M. P., Falaster, C., Fleury, M. T. L., & Fleury, A. 2017. Ownership in cross-border acquisitions and the role of government support. *Journal of World Business*, 52(4): 533-45.

Ramasamy, B., Yeung, M., & Laforet, S. 2012. China's outward foreign direct investment: Location choice and firm ownership. *Journal of World Business*, 47(1): 17-25.

Rodrigues, S. B. & Dieleman, M. 2018. The internationalization paradox: Untangling dependence in multinational state hybrids. *Journal of World Business*, 53(1): 39-51.

Rudy, B. C., Miller, S. R., & Wang, D. 2016. Revisiting FDI strategies and the flow of firm‐specific advantages: A focus on state‐owned enterprises. *Global Strategy Journal*, 6(1): 69-78.

Rugman, A. M. 1983. The comparative performance of US and European multinational enterprises, 1970-79. *Management International Review*: 4-14.

Rui, H. & Yip, G. S. 2008. Foreign acquisitions by Chinese firms: A strategic intent perspective. *Journal of World Business*, 43(2): 213-26.

Shi, W., Hoskisson, R. E., & Zhang, Y. A. 2016. A geopolitical perspective into the opposition to globalizing state‐owned enterprises in target states. *Global Strategy Journal*, 6(1): 13-30.

Sun, P., Deng, Z., & Wright, M. 2020. Partnering with Leviathan: The politics of innovation in foreign-host-state joint ventures. *Journal of International Business Studies*: 1-26.

Terjesen, S., Hessels, J., & Li, D. 2016. Comparative international entrepreneurship: A review and research agenda. *Journal of Management*, 42(1): 299-344.

Tüselmann, H.-J., McDonald, F., & Thorpe, R. 2006. The emerging approach to employee relations in German overseas affiliates: A role model for international operation? *Journal of World Business*, 41(1): 66-80.

Vasudeva, G., Nachum, L., & Say, G.-D. 2018. A Signaling Theory of Institutional Activism: How Norway’s Sovereign Wealth Fund Investments Affect Firms’ Foreign Acquisitions. *Academy of Management Journal*, 61(4): 1583-611.

Vernon, R. 1979. The product cycle hypothesis in a new international environment. *Oxford Bulletin of Economics and Statistics*, 41(4): 255-67.

VOSViewer. 2019. Welcome to VOSViewer.In VOSViewer, (Ed.): VOSViewer.

Wan, K.-M. & Wong, K.-f. 2009. Economic impact of political barriers to cross-border acquisitions: an empirical study of CNOOC's unsuccessful takeover of Unocal. *Journal of Corporate Finance*, 15(4): 447-68.

Wang, C., Hong, J., Kafouros, M., & Wright, M. 2012. Exploring the role of government involvement in outward FDI from emerging economies. *Journal of International Business Studies*, 43(7): 655-76.

Wang, C., Kafouros, M., Yi, J., Hong, J., & Ganotakis, P. 2020. The role of government affiliation in explaining firm innovativeness and profitability in emerging countries: Evidence from China. *Journal of World Business*, 55(3): 101047.

Wei, T., Clegg, J., & Ma, L. 2015. The conscious and unconscious facilitating role of the Chinese government in shaping the internationalization of Chinese MNCs. *International Business Review*, 24(2): 331-43.

Wright, M., Wood, G., Musacchio, A., Okhmatovskiy, I., Grosman, A., & Doh, J. P. 2021. State capitalism in international context: Varieties and variations. *Journal of World Business*, 56.

Xie, Z. & Li, J. 2017. Selective imitation of compatriot firms: Entry mode decisions of emerging market multinationals in cross-border acquisitions. *Asia Pacific Journal of Management*, 34(1): 47-68.

Zhang, J. & He, X. 2014. Economic nationalism and foreign acquisition completion: The case of China. *International Business Review*, 23(1): 212-27.

Zhang, J., Zhou, C., & Ebbers, H. 2011. Completion of Chinese overseas acquisitions: Institutional perspectives and evidence. *International Business Review*, 20(2): 226-38.

Zhou, N. 2018. Hybrid State-Owned Enterprises and Internationalization: Evidence from Emerging Market Multinationals. *Management International Review*, 58(4): 605-31.

Zutshi, R. K. & Gibbons, P. T. 1998. The internationalization process of Singapore government-linked companies: A contextual view. *Asia Pacific Journal of Management*, 15(2): 219-46.
